# Early-life bisphenol A exposure causes neuronal pyroptosis in juvenile and adult male rats through the NF-κB/IL-1β/NLRP3/caspase-1 signaling pathway: exploration of age and dose as effective covariates using an in vivo and in silico modeling approach

**DOI:** 10.1007/s11010-024-05039-4

**Published:** 2024-06-28

**Authors:** Ahmed S. Al-Shami, Heba-Tallah Abd Elrahim Abd Elkader, Nermine Moussa, Amina E. Essawy, Medhat Haroun

**Affiliations:** 1 Biotechnology Department, Institute of Graduate Studies and Research, Alexandria University, Alexandria, Egypt; 2 Zoology, Biological and Geological Sciences Department, Faculty of Education, Alexandria University, Alexandria, Egypt; 3 Zoology Department, Faculty of Science, Alexandria University, Alexandria, Egypt

**Keywords:** Early BPA exposure, Anxiety, Neuroinflammation, Juvenile and adult ages, Pyroptosis, NF-_k_B/IL-1β/NLRP3/Caspase-1 pathway

## Abstract

Bisphenol A (BPA), a common endocrine-disrupting chemical, is found in a wide range of home plastics. Early-life BPA exposure has been linked to neurodevelopmental disorders; however, the link between neuroinflammation, pyroptosis, and the development of psychiatric disorders is rarely studied. The current study attempted to investigate the toxic effect of BPA on inflammatory and microglial activation markers, as well as behavioral responses, in the brains of male rats in a dose- and age-dependent manner. Early BPA exposure began on postnatal day (PND) 18 at dosages of 50 and 125 mg/kg/day. We started with a battery of behavioral activities, including open field, elevated plus- and Y-maze tests, performed on young PND 60 rats and adult PND 95 rats. BPA causes anxiogenic-related behaviors, as well as cognitive and memory deficits. The in vivo and in silico analyses revealed for the first time that BPA is a substantial activator of nuclear factor kappa B (NF-κB), interleukin (IL)-1β, -2, -12, cyclooxygenase-2, and the NOD-like receptor family pyrin domain-containing 3 (NLRP3) inflammasome, with higher beclin-1 and LC3B levels in BPA rats' PFC and hippocampus. Furthermore, BPA increased the co-localization of caspase-1 immunoreactive neurons, as well as unique neurodegenerative histopathological hallmarks. In conclusion, our results support the hypothesis that neuroinflammation and microglial activation are involved with changes in the brain after postnatal BPA exposure and that these alterations may be linked to the development of psychiatric conditions later in life. Collectively, our findings indicate that BPA triggers anxiety-like behaviors and pyroptotic death of nerve cells via the NF-κB/IL-1β/NLRP3/Caspase-1 pathway.

**Supplementary Information:**

The online version contains supplementary material available at 10.1007/s11010-024-05039-4.

## Introduction

Brain development is considered an organized, but constantly adaptive, process which could be influenced by variable signals that allow neurons to undergo differentiation, migration and establishment of correct connections [1]. Early-life is a critical and very susceptible time frame because it influences postnatal outcomes. Early-life chemical exposure has been identified as a risk factor for abnormal brain development and behavioral abnormalities [2]. Endocrine disruptors are defined as natural or synthetic, usually environmental contaminants, that influence the endocrine system when exposed to humans from a variety of sources such as the environment, drinking water, dust, and food, primarily through breathing and food routes, acting to block endogenous hormones and thus disrupting normal hormonal homeostasis, behavior, and reproduction [3].

Bisphenol A (BPA) is an endocrine hormone-like disruptor that is widely utilized in the manufacturing of epoxy resins and polycarbonate plastics. In 2023, 7.49 million metric tons of BPA were produced globally, with production expected to increase to 10.13 million metric tons by 2024 [4]. Once absorbed into living organisms, BPA disrupts the hormonal system and harms human and wildlife development, cardiovascular, immunological, metabolic, neurological, and reproductive systems [5]. These negative effects may be observed after long periods of exposure and may have consequences for the next generation, i.e., they may vary depending on the time of exposure and the hormonal balance of the exposed individual, which is determined by age, sex, and other factors [6]. BPA can enter the brain through the blood–brain barrier, causing abnormal behavior in children such as cognitive impairments, attention-deficit/hyperactivity disorder (ADHD), autism spectrum disorders, anxiety, and depression [7].

Anxiety is an emotional condition characterized by increased attentiveness and increased sensory sensitivity [8]. The prefrontal cortex (PFC) is involved in a variety of cognitive tasks that support executive function, such as decision-making, working memory, and temporal processing. The hippocampus and subiculum, on the other hand, are involved in a variety of processes, including memory, emotional processing, spatial and contextual processing [9]. The hippocampus-to-PFC pathway is the primary efferent anatomical connection from the hippocampal formation to the PFC. This pathway's neuronal activity is very susceptible to stress from various sources, which is a primary triggering factor for symptoms of anxiety disorders [9]. Aside from the implication of a reciprocal effect of both environmental and genetic factors, a multitude of evidence suggests that neuroinflammation plays a critical role in the etiology and maintenance of anxiety disorders. Neuroinflammatory events are triggered when resident immunocompetent cells (astrocytes and microglia) detect pathogen-associated molecular patterns (PAMPs) or damage-associated molecular patterns (DAMPs) [10, 11].

Autophagy is a major homeostasis-maintaining function in living cells [12]. It is regulated by proteins involved in endosomal/phagosomal pathways, as well as a network of proteins encoded by autophagy-related genes, such as Atg8 (LC3), which is often employed as a marker for quantifying and detecting autophagosomes [13]. Moreover, pyroptosis is defined as a lytic and caspase-dependent pro-inflammatory form of regulated cell death that is produced by inflammasomes and conducted by various caspase family members and the gasdermin protein family [14]. The most promiscuous sensor, the Nod-like receptors family pyrin domain containing 3 (NLRP3), is capable of detecting a wide range of damage or stress signals. It binds to the adaptor protein apoptosis-associated speck-like protein (ASC), which recruits pro-caspase-1 to build the inflammasome protein complex [15]. Caspases-1 matures as a result of inflammasome activation and subsequently transforms pro-forms of interleukin (IL)-18 and IL-1β into their active forms, causing pro-inflammatory responses [16]. Both inflammasome and autophagy-related proteins have been genetically linked to inflammatory illnesses, including neuropsychiatric pathologies, implying that these two processes are functionally linked [17].

In the present study, we focused on the effect of BPA on inflammatory cytokines, the NLRP3 inflammasome activation and subsequent stimulation of pyroptosis, added to histological alterations in the PFC and hippocampus (dentate gyrus) of postnatal rats exposed to BPA. For the first time, we discussed how environmental BPA exposure at different doses in juvenile and adulthood ages can induce pyroptotic death of nerve cells via the IL-1β/ NF-κB/NLRP3/Caspase-1 signaling pathway, and how this contributes to the development of anxiety-like behaviors, memory and cognitive impairments.

## Materials and methods

### Experimental animals and housing

Twenty sexually mature female Sprague Dawley (SD) rats weighing 200–210 g were purchased from Alexandria University's Faculty of Medicine. Rats were housed in polycarbonate cages under pathogen-free conditions in a temperature (25 ± 2 °C) and humidity-controlled (50 ± 10%) environment on a 12 h light/dark cycle (lights on at 07:00). Starting at 21:00 h, two females were held overnight with one sexually mature male for mating purposes. Females were tested for the existence of a vaginal plug, which represented gestational day zero, by 7:00 a.m. the next morning. Pregnant females were housed separately, fed a standard rat diet, and given free access to tap water until their young were weaned (PND18). Male pups weighing between 25 and 38 g at PND18 were chosen for the study. All animal experiments were performed according to the National Institutes of Health Guide for the Care and Use of Laboratory Animals. Experimental protocols and animal handling procedures were approved by Alexandria University Institutional Animal Care and Use Committee (ALEXU-IACUC), a member of the International Council for Laboratory Animal Science (ICLAS) (Approval number: AU14-22-1003-2-5).

### Experimental design

Fifty-four juvenile male SD rats (PND18) were randomly assigned to one of six groups. Rats from the first and second groups (n = 6 for each group) acted as controls and were given 0.5 mL of corn oil orally. The third and fourth group (n = 6 for each group) of rats was administered BPA (98%; CAS: 80-05-7; Loba Chemie for Laboratory Reagents and Fine Chemicals, India) orally at a dose of 50 mg/kg/day dissolved in corn oil until PND 60 and PND 95, respectively [18, 19]. Cohorts of the fifth and sixth groups (n = 6 for each group) contained rats orally given with BPA at dosages of 125 mg/kg/day until PND 60 and PND 95, respectively.

### Behavioral studies

Behavioral testing was carried out from PND 46 to PND 58 for the juvenile age phase (PND 60) and from PND 81 to PND 93 for the adulthood age phase (PND 95). The active time for starting behavioral tasks was between 9:00 A.M. to 2:00 P.M. with a 24-h rest period between tests to minimize stressful conditions. The duration of daily testing was balanced throughout the various experimental groups. Animal groups and animals were counterbalanced between and within tested rat cohorts to minimize possible negative influences of daytime. Rats were acclimatized to the testing environment for at least 15 min before testing. The activities of the animals during behavioral tests were recorded using a video camera linked to a computer. Between subjects, each testing instrument was cleaned with a 70% ethanol solution.

### Open field test (OFT)

The OFT apparatus is used to assess rat exploration, anxiety level, and motor activity. The apparatus was made up of a white square wooden frame with opaque walls that measured 100 × 100 × 50 cm. The floor was divided into 16 equal-sized (4 × 4 cm) squares, and the test was conducted in a quiet and dimly illuminated room [20]. At the start of the test, each animal was separately placed on the periphery and permitted to freely explore the OFT apparatus for 5 min. (1) The number of laps, (2) the distance in the central zone, (3) the distance in the peripheral zone, (4) the total distance, (5) the number of entries into the central zone, (6) the number of entries into the peripheral zone, and (7) the time in the central zone/total time were recorded [21, 22].

### Elevated plus maze (EPM)

The EPM was used to assess the male rat's anxiety-like behavior, as well as the tendency for learning and memory retention. A plus-shaped maze with two opposed open arms (50 cm in length × 5 cm in width) and two opposite closed arms (50 cm in length × 5 cm in width, and 25 cm in height) extending out from a central platform (5 cm × 5 cm) was employed. Each rat was placed at the end of one of the open arms, facing away from the central platform, and was free to explore the open and closed arms for 5 min. During this experiment, each rat's (1) closed arm distance, (2) open arm distance, (3) total distance, (4) time in the open arm/total time, and (5) duration to reach the exit end were recorded [22, 23].

### Y-maze test

The Y-maze test measures exploratory behavior, learning, and short-term spatial memory. Three identical arms (50 cm in length, 20 cm in width, and 25 cm in height) were linked in the middle to make a “Y” shape. All of the arms’ walls were 8 cm height, allowing the rat to observe distal spatial landmarks. Home, novel, and familiar were assigned at random to the three arms. In brief, each rat was placed in the home arm (start arm) and given 10 min to explore both the home and familiar arms (training trial). After a 24-h intertrial delay, each rat was returned to the maze and permitted to freely explore all three arms for 5 min. For each rat, the following parameters were recorded: (1) percentage of novel arm visits, (2) percentage of home arm visits, (3) percentage of familiar arm, (4) percentage of duration in the novel arm, (5) percentage of duration in the home arm, (6) percentage of duration in the familiar arm, (7) frequency of alternate arm returns, and (8) total arm entries [23].

### Brain tissue preparation

Rats were fasted overnight at the end of the experiment's dual phases, as described above. The period of overnight fasting before sampling didn’t cause any remarkable loss of body weight or observed adverse effects. Rats were anaesthetized by intraperitoneal injection of xylazine and ketamine (5 mg/kg + 100 mg/kg), followed by cervical dislocation. All rats' brains were quickly removed, weighed, rinsed in cold normal saline, and dissected to isolate the PFC and hippocampus. The isolated brain regions of six rats from each group were stored at − 80 °C until used for biochemical analysis. Using a tissue homogenizer, the hippocampus and PFC regions were homogenized in a 20 times (w/v) ice-cold TES buffer (25 mM Tris–HCl, 0.2 mM EDTA, and 0.33 M sucrose, pH 7.4) with a protease inhibitor cocktail (SRE0055, Sigma-Aldrich, Germany) and then incubated at 4 °C for 1 min while shaking. Supernatants were recovered after centrifuging the homogenates at 1800×*g* for 10 min at 4 °C (Hettich zentrifugen, Universal 32 R; Hettich Lab. Tuttlingen, Germany).

For immunohistochemical and histological examination, three rats from each group were perfused with paraformaldehyde 4% and sacrificed. PFC and hippocampus were isolated and quickly fixed in 10% formalin.

### Biochemical analyses

#### Neuroinflammatory markers

Neuroinflammatory markers in the PFC and hippocampus were measured using commercially available enzyme-linked immunosorbent assay (ELISA) kits. NF-κB (Biosource, cat no. MBS453975), IL-1β (Biosourse, cat no. MBS825017), IL-2 (Abcam, ab221834), IL-12 (Cusabio, CSB-E07362r), and COX-2 (Cusabio, CSB-E13399r). Each assay was performed following the kit makers' instructions.

#### Autophagy markers

NLRP3-inflammasome levels, as well as LC3A, LC3B and beclin-1 autophagic markers, were measured in the PFC and hippocampus using commercially available ELISA kits. Fine Test (cat no. ER1965) provided the NLRP3 kit, LSBio (cat no. LS-F9917; LS-F19802) provided the LC3A and LC3B kits, respectively, and Cusabio (cat no. CSB-EL002658RA provided the beclin-1 kit. Each assay was performed following the kit makers' instructions. A microplate ELISA reader was used to measure the optical density at 450 nm.

### Histopathological examination

The formalin-fixed brain tissues (PFC and DG) were dehydrated with ethanol alcohol in ascending grades, cleaned in xylene, and then embedded in paraffin wax for 2 h. The paraffin-embedded brain tissues were sectioned serially at 5 μm, de-waxed, hydrated, and stained with hematoxylin and eosin (H&E). The PFC and DG were examined using a light microscope (Olympus LC20, Germany), and images were obtained using a digital camera attached to the microscope. The morphometric analysis of H&E staining data was completed using Image J analyzer (version 5.1) by counting the number of granular cells in both PFC and DG, the number of pyramidal cells in the PFC, the thickness of granular cells in DG, angiectasis, the number of apoptotic cells, and the relative area of neuroinflammation and neuronal loss in both brain regions in eight repetitions from each specified brain region on each animal’s section [24].

### Immunohistochemical examination of caspase-1 immunoreactive cells

The paraffin-embedded brain sections (PFC and DG) were mounted on poly-l-lysin-coated slides and incubated overnight on the benchtop at 37 °C. After that, the paraffin sections were deparaffinized and hydrated in the following order: 10 min in xylene twice; 5, 10, 10, and 10 min in 100% absolute, 95%, 85%, and 70% ethanol alcohol, and 5 min in PBS at room temperature three times. After 30 min of incubation with Ultra V Block reagent, the sections were incubated overnight at 4 °C with caspase-1 primary antibody. Finally, the sections were incubated for 30 min at room temperature with UltraVision ONE HRP Polymer. The peroxidase reaction was visualized in the dark for 7–9 min at room temperature using a 3,30-diaminobenzidine (DAB) chromogen mixture. Sections were counterstained with hematoxylin. Immunostaining was achieved by incubating cells with an anti-caspase-1 antibody; positive cells had brownish-yellow cytoplasm. Using the Image J analyzer (version 5.1), eight fields from each brain section were selected and examined for positive caspase-1 immunostaining intensity [25].

### Molecular docking

To predict whether BPA could directly interact with different molecules involved in neuroinflammation, autophagy, and pyroptosis, molecular docking analysis of BPA and those molecules was performed. The three-dimensional chemical structure of BPA was obtained from the PubChem Open Chemistry Database (NIH, USA, https://pubchem.ncbi.nlm.nih.gov), and protein structures were obtained from the RCSB Protein Data Bank (PDB) (https://www.rcsb.org/). The processed BPA and ligand structures were then converted to pdbqt files for molecular docking analysis. The criteria for selecting the structure of target molecules were as follows: (i) the structure was discovered by X-ray diffraction, and (ii) the resolution was less than 3 Angstrom. Structures of water and heteroatoms surrounding target proteins were removed, and all missing polar hydrogens and Kollman charges were added to the target proteins using AutoDockTools 4.2 software [26]. The processed target protein structures were then converted to pdbqt files.

Procedures for molecular docking analysis were carried out as previously described by Kanlayaprasit et al. [27]. Briefly, rigid docking of the target proteins with BPA was performed using AutoDockTools 4.2 software with the Lamarckian GA algorithm and default parameters recommended by the software. For each run, the docking parameter file was generated by utilizing Lamarckian GA docking parameters as follows: number of GA runs: 50, population size: 300, maximum number of evaluations: 2,500,000, gene mutation rate of 0.02, number of generations: 27,000, and cross-over rate: 0.8 [28]. The most favorable docking poses were represented by the binding affinity (ΔG). Subsequently, the root means square deviation (RMSDb), the inhibition constant (Ki), and the molecular interaction of the selected poses were evaluated. The structures of ligands, proteins and docking complexes were visualized using the Biovia Discovery Studio 3.0 software.

### Statistical analysis

The statistical analysis was carried out using the Statistical Package for the Social Sciences (SPSS, version 16.0) software. The data from six determinations were expressed as mean values ± SEs. Each data set was checked for normality of distribution and homogeneity of variances using the Shapiro-Wilks and the Levene tests, respectively. Data showing normal distribution were analyzed with multivariate analysis of variance (MANOVA), followed by the multi-comparison Tukey’s test. A level of P < 0.05 was considered significant. GraphPad Prism 8.0 software (USA) was used to draw all of the figures.

The Euclidean distance similarity matrix was calculated using a matrix including the inflammatory and autophagy descriptors for each dose and age. Before being submitted to Principal Coordinates Analysis (PCO), this matrix was simplified by computing the distance between centroids based on the groups (control, BPA 50–60, BPA 50–95, BPA 125–60, and BPA 125–95). A GGE biplot for data analysis and visualization was used to confirm the PCO of indicators with substantial differences across groups. Pearson's correlation coefficient was utilized to determine any correlations between variables.

## Results

### Behavioral studies

#### OFT

**Fig. 1:**
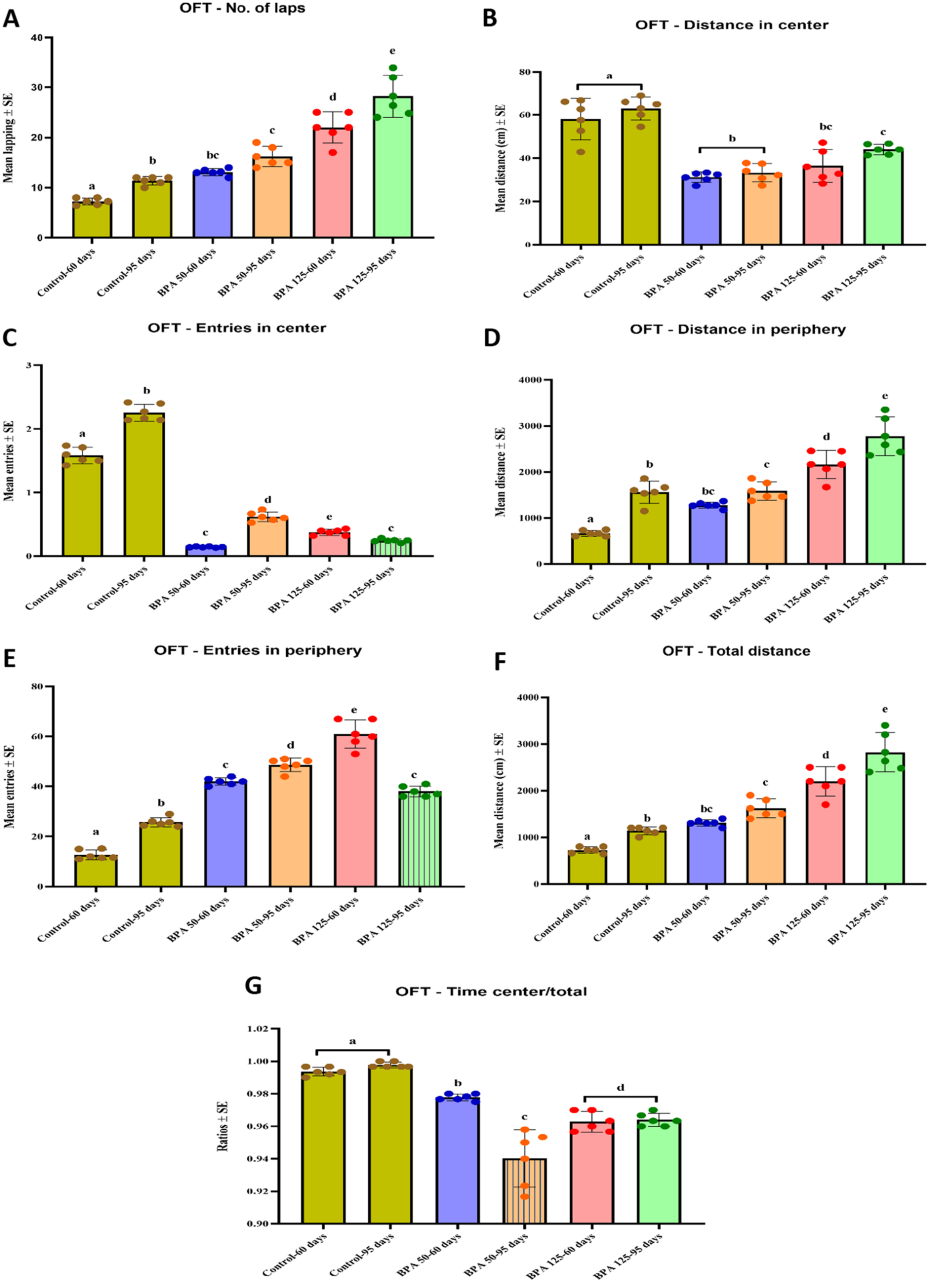
Effect of postnatal BPA exposure on the performance of PND60 and PND95 male rats in the OFT **A** number of laps, **B** Distance in the center, **C** Distance in the periphery, **D** Total distance, **E** Number of entries in center zone, **F** Number of entries in periphery, **G** Time center/total time The results are expressed as the mean ± SE (n = 6 rats/group). Groups with different superscript letters means there is a significant change between them at P < 0.05. Statistical significance test for comparison was done by MANOVA, followed by post hoc Tukey’s HSD multiple comparison test

Postnatal exposure to BPA significantly (P < 0.05 vs. control) increased the number of laps in both PND 60 and PND 95 male rats in the OFT (F_(5,35)_ = 69.300, Fig. 1A). Furthermore, the distance travelled and the number of entries to the center zone in BPA-treated rats were significantly lower in all BPA-treated groups when compared to the controls (F(_5,35)_ = 33.834, 664.437, respectively, Fig. 1B, C). However, postnatal BPA exposure significantly (P < 0.05 vs. control) increased the distance travelled and number of entries to the periphery (F_(5,35)_ = 71.827, 213.756, respectively, Fig. 1D, E). Moreover, BPA administration significantly (P < 0.05 vs. control) increased total distance (F_(5,35)_ = 69.300, Fig. 1F) while decreasing the time spent in the core zone/total time (F_(5,35)_ = 47.661, Fig. 1G).

#### EPM

**Fig. 2:**
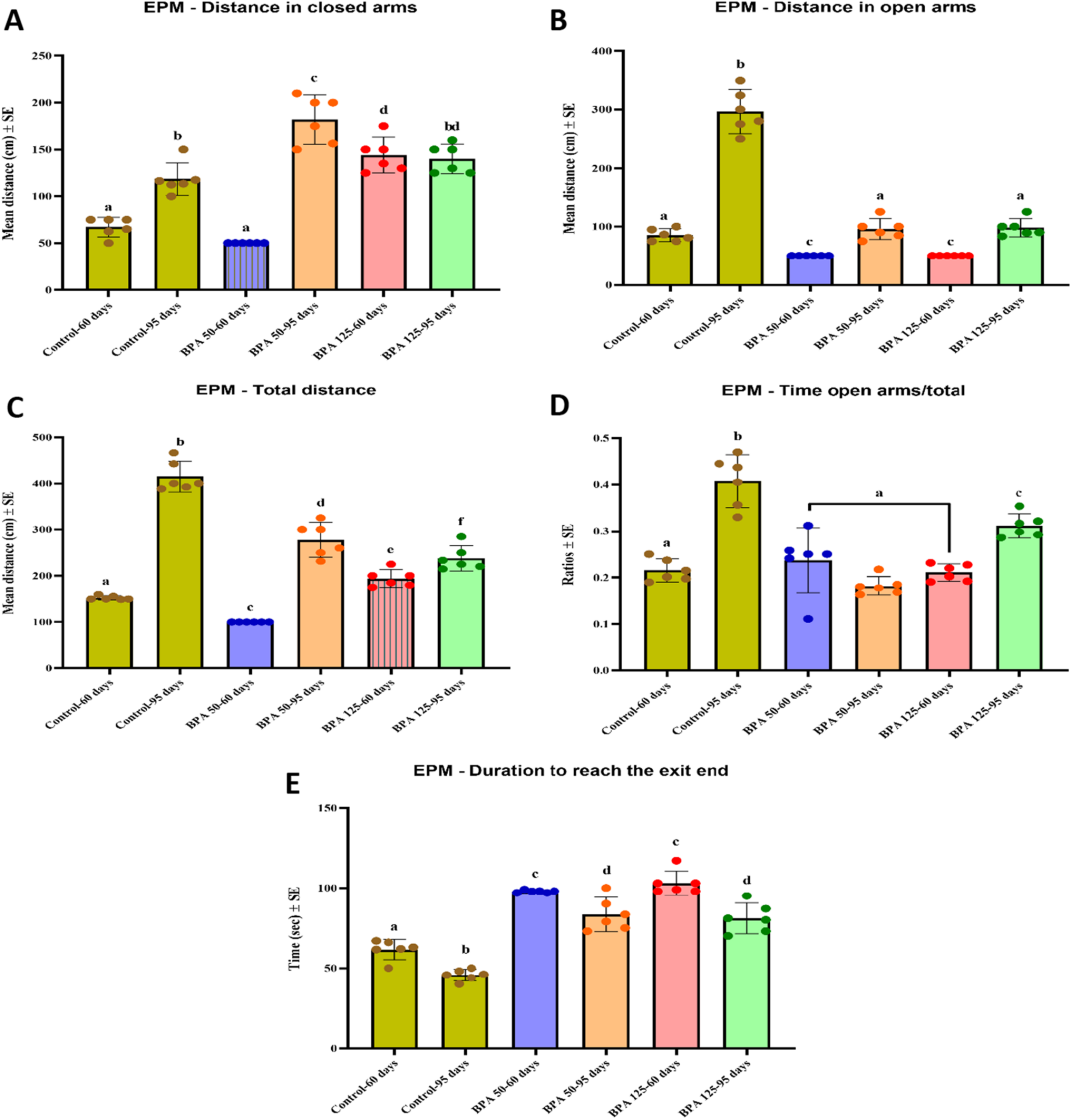
Effect of postnatal BPA exposure on the performance of PND60 and PND95 male rats in the EPM. **A** Distance in the closed arm, **B** distance in the open arm, **C** total distance, **D** time in the open arm/total time, **E** duration to reach the exit end. The results are expressed as the mean ± SE (n = 6 rats/group). Groups with different superscript letters means there is a significant change between them at P < 0.05. Statistical significance test for comparison was done by MANOVA, followed by post hoc Tukey’s HSD multiple comparison test

Postnatal BPA exposure increased the distance in the closed arm in all BPA-treated groups except the PND 60 group treated with 50 mg/kg/day, which showed a significant decrease compared to the control (F_(5,35)_ = 56.929, Fig. 2A). The distance in the open arm, on the other hand, was found to be significantly (P < 0.05 vs. control) decreased in all BPA-exposed groups (F_(5,35)_ = 159.738, Fig. 2B). Furthermore, the total distance was found to be considerably lower in all BPA-treated groups, with the exception of the PND 60 group treated with 125 mg/kg/day, which showed a significant increase in comparison to controls (F_(5,35)_ = 130.955, Fig. 2C). The time spent in the open arm in rodents is described as having an anxiolytic effect. BPA did not create significant changes in the time spent in the open arm in groups of PND 60, but it did cause a significant decrease in those with PND 95 postnatal exposure (F_(5,35)_ = 27.415, Fig. 2D). Moreover, BPA exposure resulted in a significant increase in the duration to reach the exit end when compared to the control groups (F_(5,35)_ = 59.087, Fig. 2E).

#### Y-maze

**Fig. 3:**
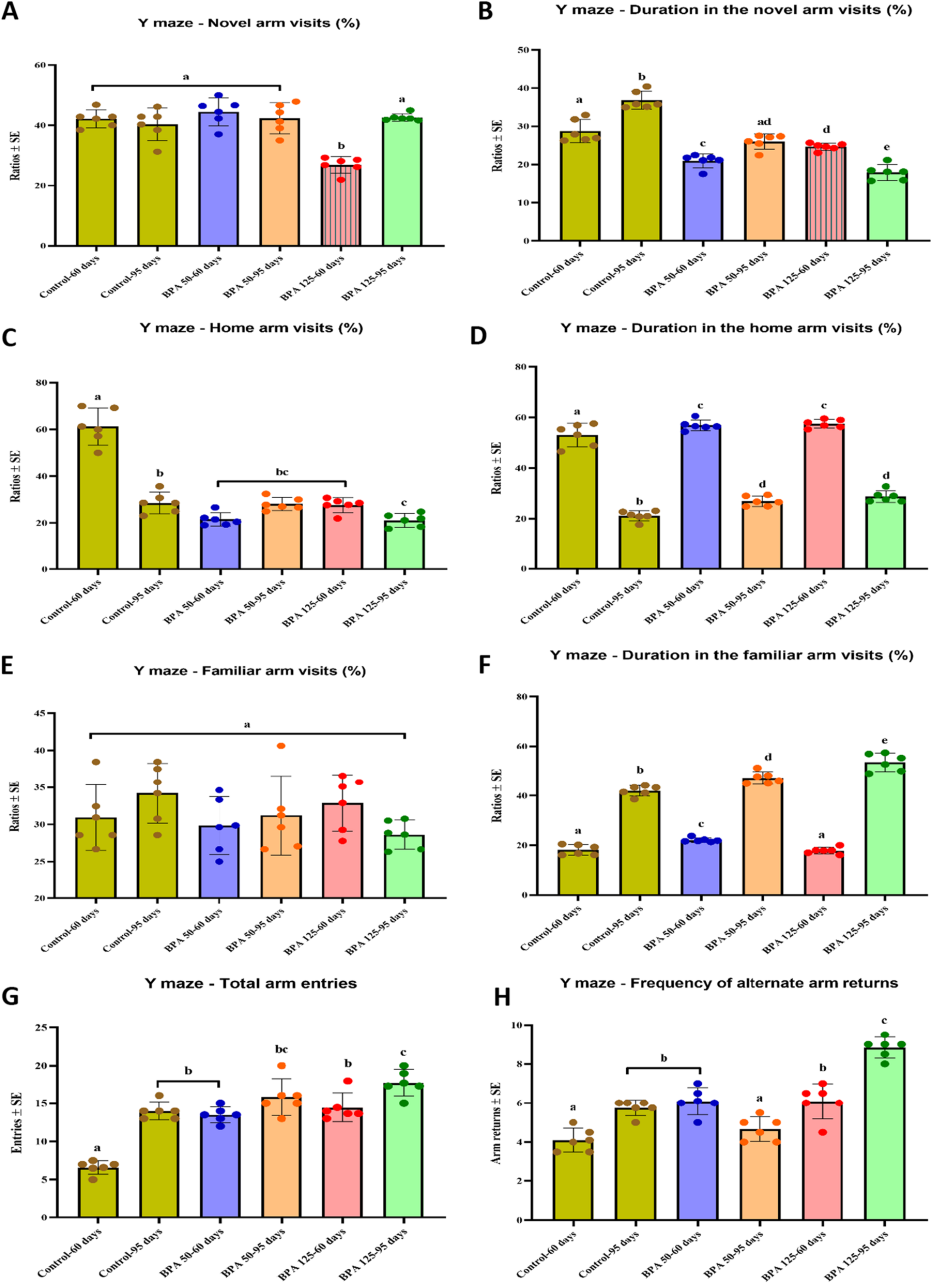
Effect of postnatal BPA exposure on the on the behavioral performance of PND60 and PND95 male rats in the Y-maze test. **A** Novel arm visits %, **B** duration in the novel arm %, **C** home arm visits %, **D** duration in the home arm, **E** familiar arm visits %, **F** duration in the familiar arm %, **G** total arm entries, **H** frequency of alternate arm returns. The results are expressed as the mean ± SE (n = 6 rats/group). Groups with different superscript letters means there is a significant change between them at P < 0.05. Statistical significance test for comparison was done by MANOVA, followed by post hoc Tukey’s HSD multiple comparison test

The Y-maze test is used to assess rats’ short-term spatial memory based on their tendency to explore novel locations. Our results demonstrated that postnatal BPA exposure increased the percentage of novel arm visits, with the exception of the PND 60 exposed to higher dose of BPA group, which showed a significant decrease compared to the control groups (F_(5,35)_ = 17.486, Fig. 3A). Conversely, the percentage of time spent in the novel arm was shown to be significantly lower (P < 0.05 vs. control) in all BPA-exposed groups (F(_5,35)_ = 62.143, Fig. 3B). Otherwise, the percentage of visits to the home and familiar arm (Fig. 3C, E) was found to be lower in all BPA-treated rats, while the duration spent in these arms was significantly (P < 0.05 vs. control; Fig. 3D, F) increased, with the exception of PND 60 at a BPA dose of 125 in the familiar arm. Furthermore, total arm entries and AAR were found to be significantly higher in all BPA-exposed groups (F_(5,35)_ = 35.871 and F_(5,35)_ = 42.040, respectively, Fig. 3G, H).

#### Neuroinflammatory markers

**Fig. 4:**
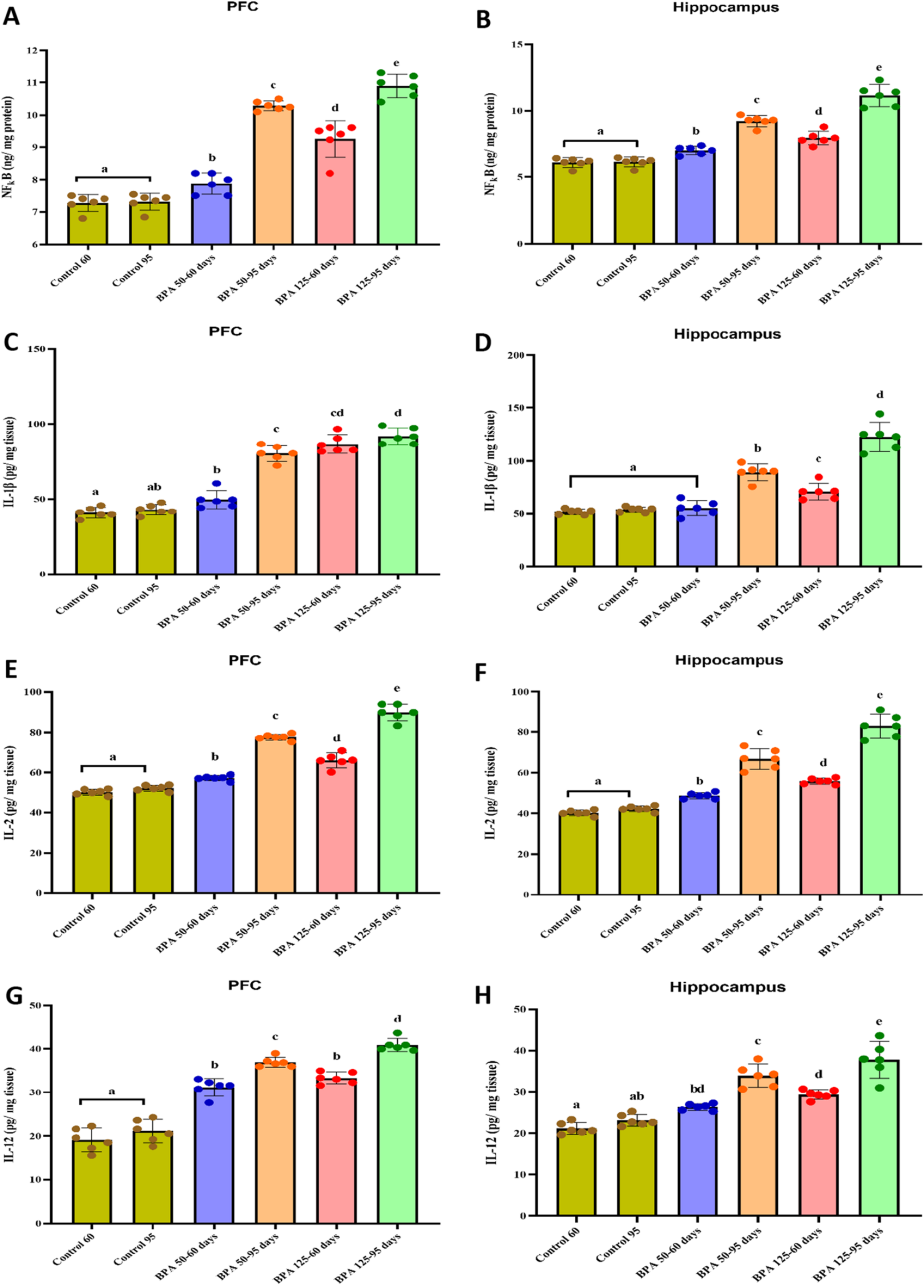

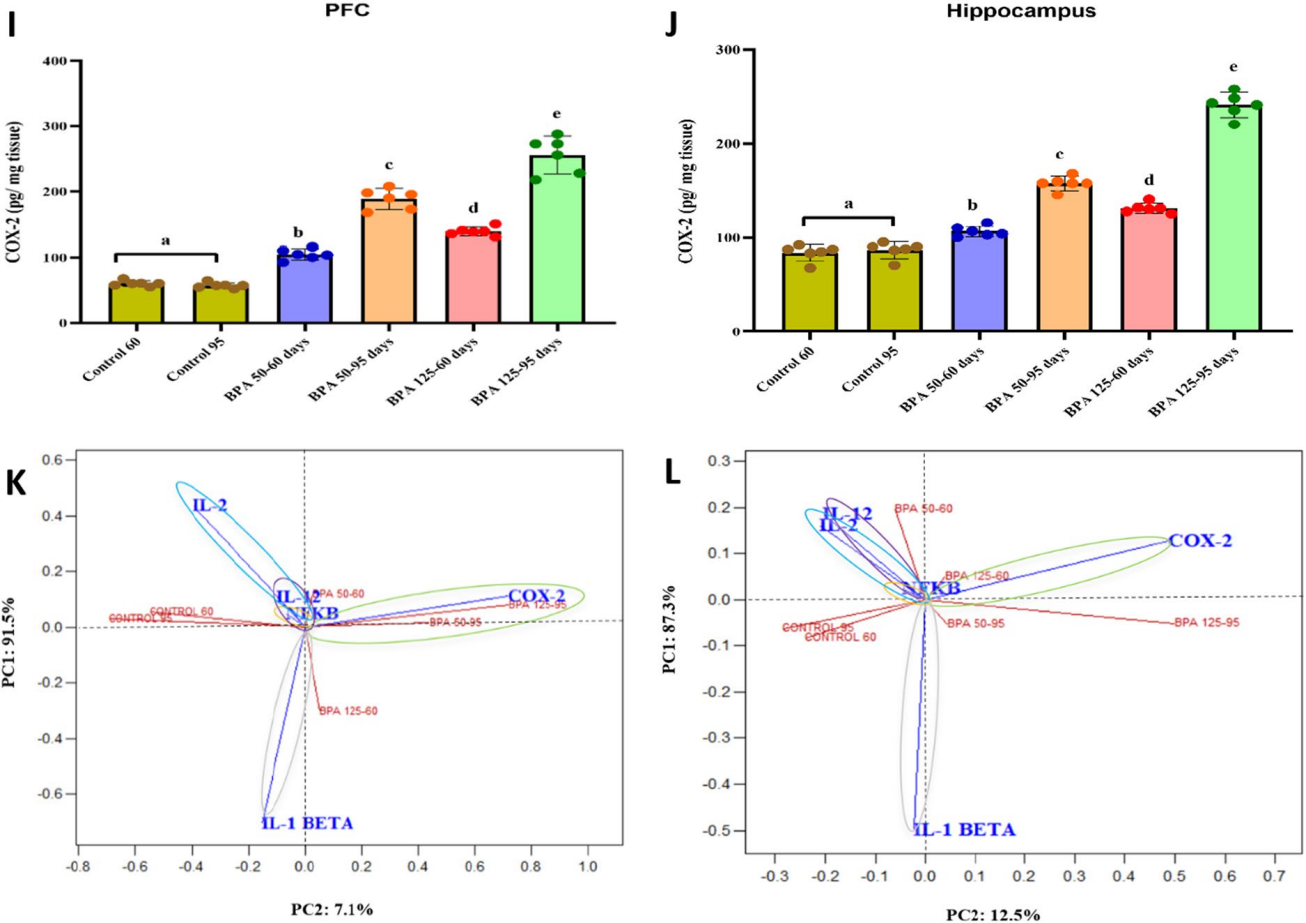
Effect of postnatal exposure to BPA (50 and 125 mg/kg/day) on the expression level of NF-kB (**A** and **B**), IL-1β (**C** and **D**), IL-2 (**E** and **F**), IL-12 (**G** and **H**), COX-2 (**I** and **J**) in the PFC and hippocampusof PND60 and PND95 male rats. Inflammatory markers for each dose and age plotted on axes 1 and 2 of a Principal Coordinates (PCO) graph by computing the distance between centroids based on the groups in the PFC and hippocampus (**K** and **L**). The results are expressed as the mean ± SE (n = 6 rats/group). Groups with different superscript letters means there is a significant change between them at P < 0.05. Statistical significance test for comparison was done by MANOVA, followed by post hoc Tukey’s HSD multiple comparison test

Figure 4 depicts the effect of BPA on inflammatory cytokines in the PFC and hippocampus of postnatal male rats. Oral treatment of BPA significantly (P < 0.05 vs. control) increased cortical and hippocampal NF-κB (F_(5,35)_ = 134.919, 100.978, respectively, Fig. 4A, B) levels. Moreover, BPA significantly elevated the expression level of IL-1β (F_(5,35)_ = 136.836, 82.820, respectively, Fig. 4C, D), IL-2 (F_(5,35)_ = 246.263, 151.838, respectively, Fig. 4E, F), IL-12 (F_(5,35)_ = 124.535, 47.423, respectively, Fig. 4G, H) and COX-2 (F_(5,35)_ = 188.592, 302.089, respectively, Fig. 4I, J) in both brain regions compared to control groups.

#### Autophagy markers

**Fig. 5:**
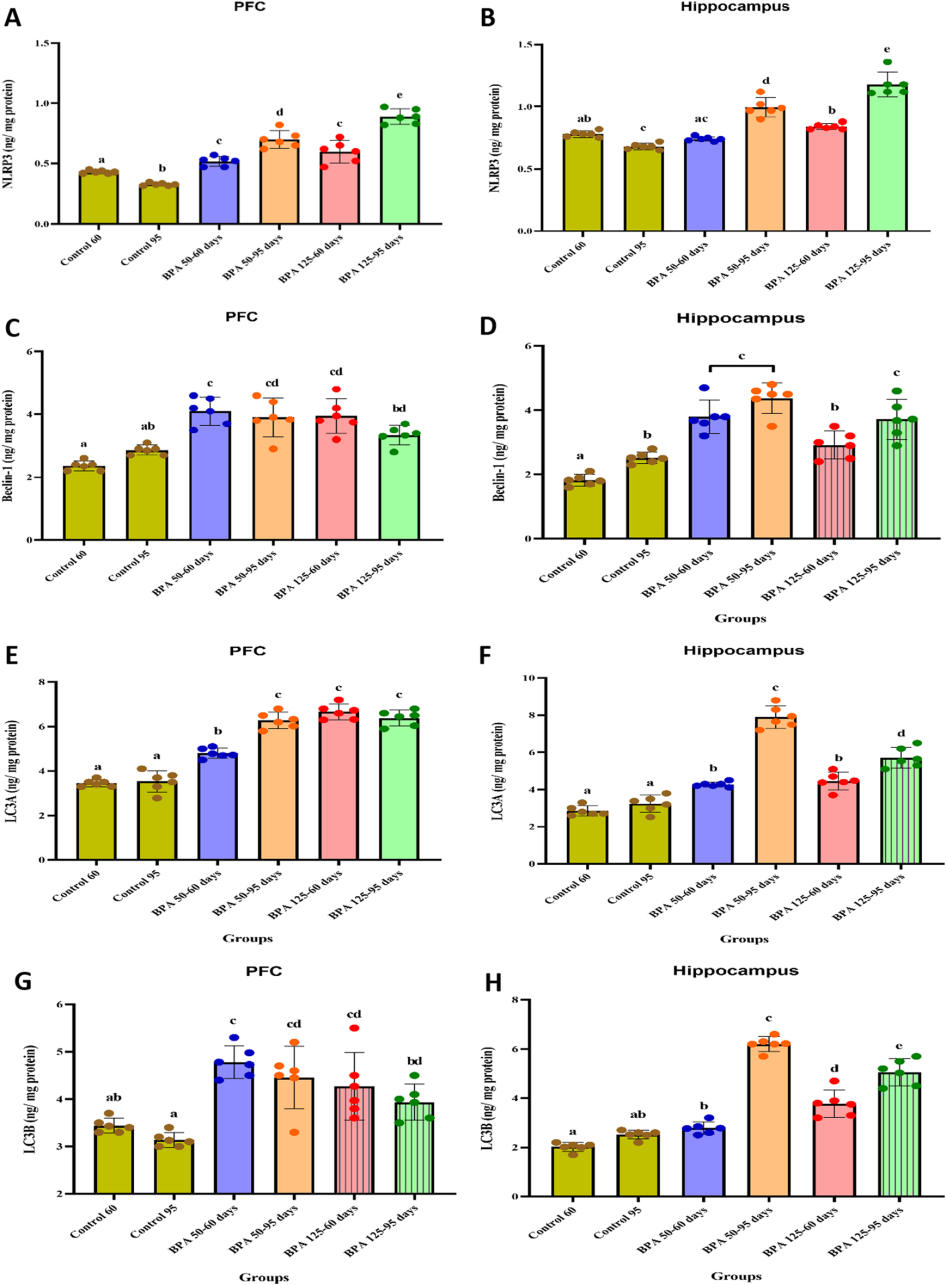

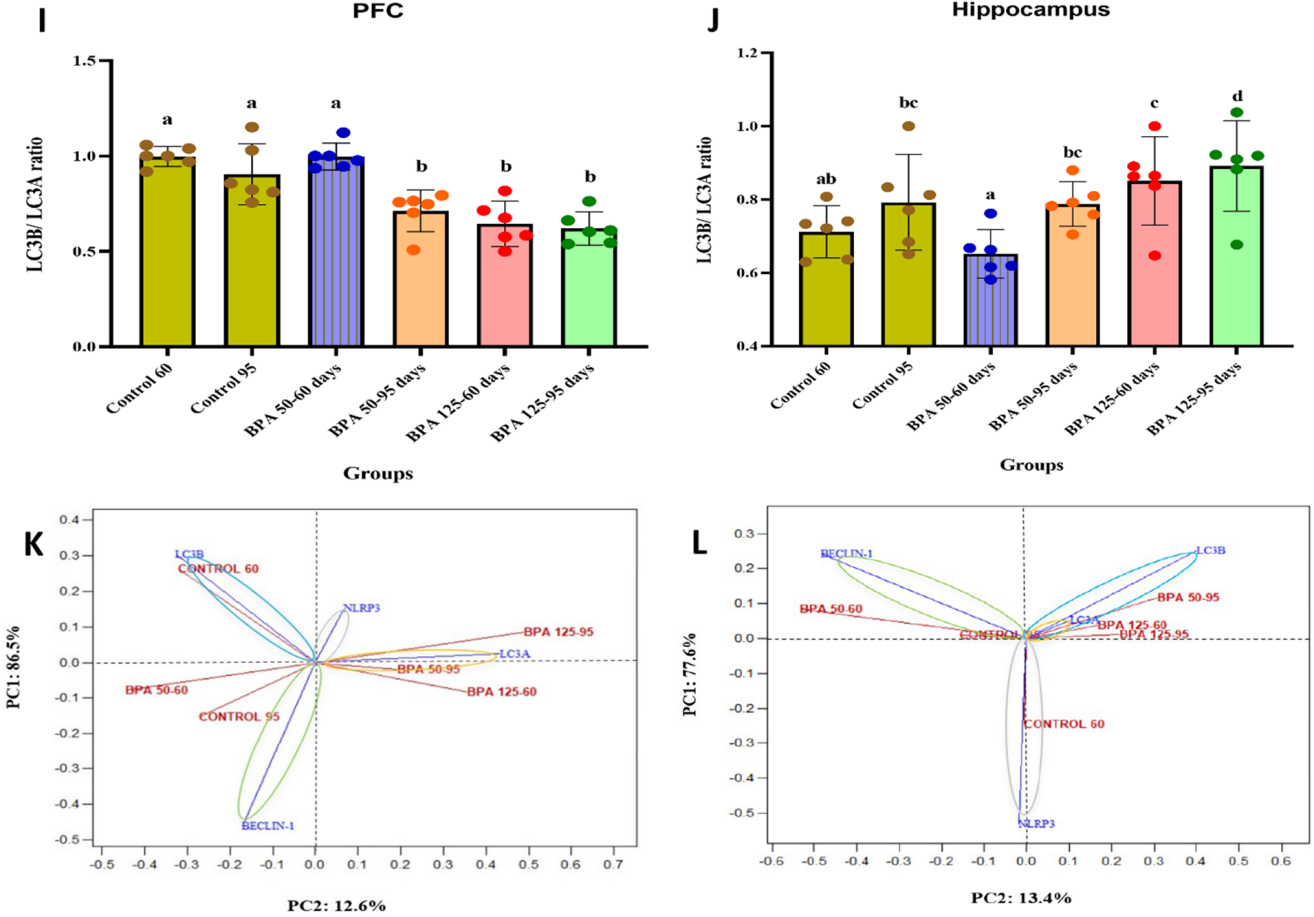
Effect of postnatal exposure to BPA (50 and 125 mg/kg/day) on the expression level of NLRP3 (**A** and **B**), Beclin-1 (**C** and **D**), LC3A (**E** and **F**), LC3B (**G** and **H**), and LC3B/LC3A ration (**I** and **J**) in the PFC and hippocampusof PND60 and PND95 male rats. Autophagy markers for each dose and age plotted on axes 1 and 2 of a Principal Coordinates (PCO) graph by computing the distance between centroids based on the groups in the PFC and hippocampus (**K** and **L**). The results are expressed as the mean ± SE (n = 6 rats/group). Groups with different superscript letters means there is a significant change between them at P < 0.05. Statistical significance test for comparison was done by MANOVA, followed by post hoc Tukey’s HSD multiple comparison test

Postnatal BPA treatment induced a significant (P < 0.05 vs. control) increase in NLRP3 levels in both the PFC and hippocampus of all groups, except for BPA 50 at PND 60 in the hippocampus (F_(5,35)_ = 76.785, 76.450, respectively, Fig. 5A, B). Furthermore, BPA caused a significant (P < 0.05) increase in cortical and hippocampal beclin-1 (F_(5,35)_ = 18.293, 30.889, respectively, Fig. 5C, D), LC3A (F_(5,35)_ = 121.178, 110.262, respectively, Fig. 5E, F) and LC3B (F_(5,35)_ = 12.342, 124.341, respectively, Fig. 5G, H) levels. Moreover, the LC3B/LC3A ratio was significantly (P < 0.05 vs. control; Fig. 5I) decreased in the PFC of all groups, whereas no significant changes showed in BPA 50–60. Otherwise, the LC3B/LC3A ratio was significantly increased in the hippocampus of BPA 125 at PND 60 and PND 95, while BPA 50 showed no significant changes at both ages (Fig. 5J).

#### Histopathological examination and morphometric analysis

**Fig. 6:**
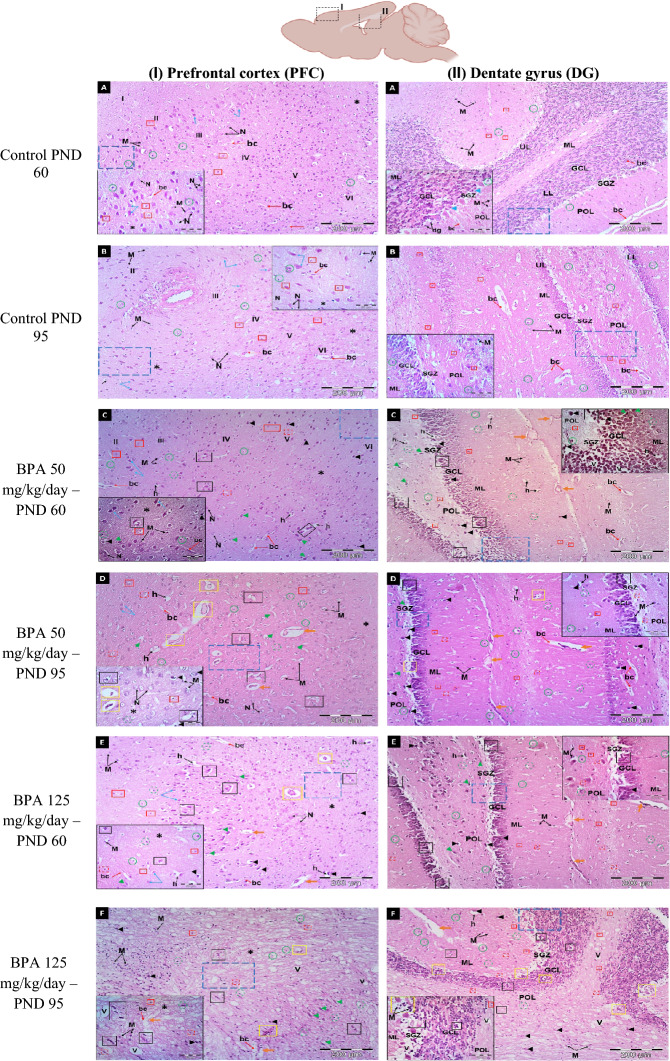

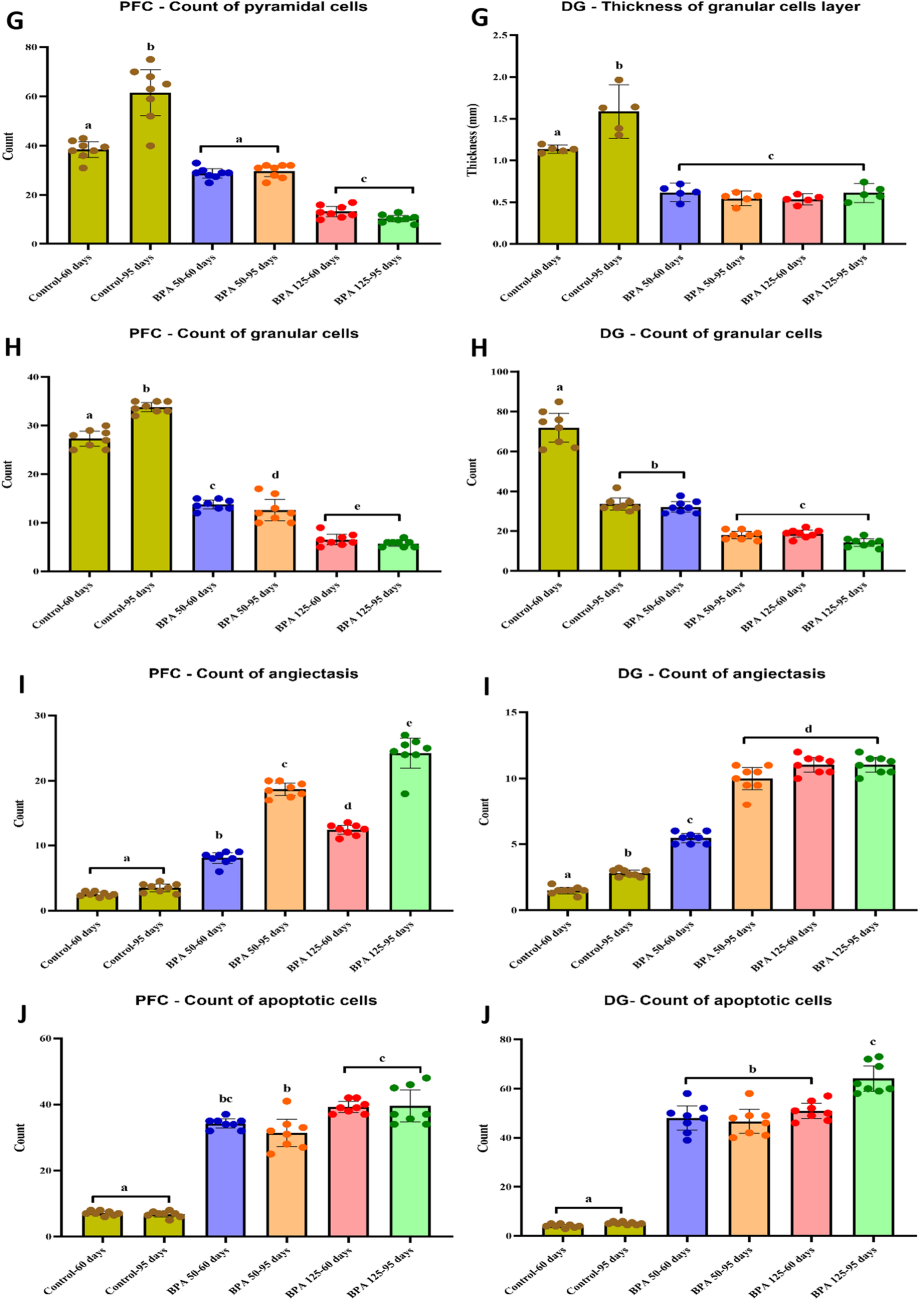

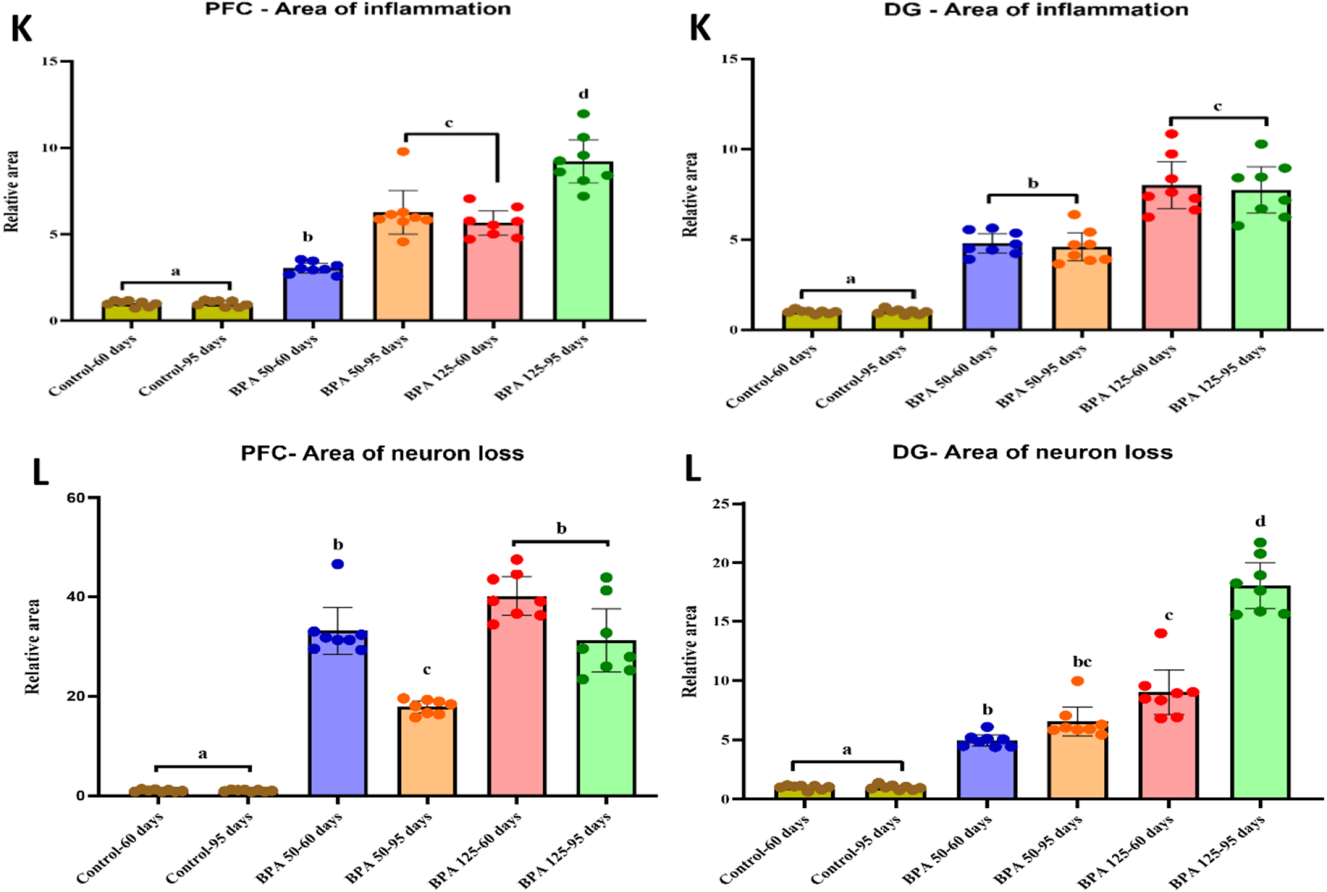
Representative photomicrograps of H&E stained coronal sections at the prefrontral cortex (PFC) and Dentate gyrus (DG) of rats in the six experimental groups: **A** Control PND 60, **B** Control PND 95, **C** BPA 50-PND 60, **D** BPA 50-PND 95, **E** BPA 125-PND 60, **F** BPA 125-PND 95, **G** count of pyramidal cells in PFC and thickness of GCL in DG, **H** count of granular cells, **I** count of angiectasis, **J** count of apoptotic cells, **K** relative area of inflammation, **L** relative area of neuronal loss. *Abbreviations and symbols I* Molecular layer**, ***II* External granular layer, *III* External pyramidal layer, *IV* Internal granular layer, *V* Internal pyramidal layer, *VI* Polymorphic (multiform) layer, *ML* molecular layer of DG, *POL* polymorphic layer, *GCL* granular cell layer, *SGZ* subgranular zone, *UL* upper limb of DG, *LL* lower limb, *bc and red arrow* blood capillary, *N* larger neuronal cell bodies with vesicular open face nuclei, *M* microglial cell, *h* pericellular halo, *V* vacuole, *asterisk* neuropile, *blue arrow* pyramidal cell, *green arrow head* flame-like cell with pointed end, *black arrow head* pyknotic cell, *orange arrow* angiectasis, *black square* neuronal degeneration, *yellow square* neurophagia, *red square* astrocyte, *dashed red square* degenerated astrocyte, *green circle* oligodendrocyte, *dashed green circle* degenerated oligodendrocyte. The blue dashed square indicated the area of magnification. The results are expressed as the mean ± SE (n = 8 fields/group). Groups with different superscript letters means there is a significant change between them at P < 0.05. Statistical significance test for comparison was done by MANOVA, followed by post hoc Tukey’s HSD multiple comparison test

The normal histological structure of the PFC region was arranged into six successive layers stratified by the outer molecular (I), followed by the external granular (II), the external pyramidal (III), the internal granular (IV), the internal pyramidal (V), and the polymorphic layer (VI) (Fig. 6A, B). The layers were characterized by neuronal cell bodies (soma) with rounded open-face nuclei with prominent nucleoli surrounded by a border of basophilic cytoplasm. These layers' neuropile seemed acidophilic, entangling a mat of glial and neuronal cell processes with regular blood capillaries. They also displayed the nuclei of different neuroglial cells.

The prefrontal cortices of PND-60 rats treated orally with BPA at a dose of 50 mg/kg/day showed the presence of many normal neuronal cell bodies as well as a few shrunken with highly pigmented pyknotic nuclei and the emergence of a few flame-like cells. A few neuroglial cells also revealed signs of degeneration (Fig. 6C). On the other hand, the PFC of rats at the age of PND-60 exposed to a higher dose of BPA (125 mg/kg) displayed conspicuous lesions including neuronal loss (pyramidal and granular cells), angiectasis (vascular dilation), and neuronophagia (clusters of activated microglial cells encircling degenerating neurons), along with the presence of flame-like cells with a pointed end and shrunken neurons with pyknotic nuclei and pericellular haloes (Fig. 6E). Notably, the prefrontal cortices of PND-95 rats showed progressive neuronal degeneration, more severe angiectasis in deeper cortical layers, a multiplicity of neurophagia sites, and disorganized and vacuolated neuropil that was accompanied by a large number of activated microglia, particularly at higher BPA doses (Fig. 6D, F).

The DG of the control groups was made up of three main layers, which were arranged as follows: molecular layer (ML), granule cell layer (GCL), which served as the primary cell layer, and finally, polymorphic layer (POL), which formed the DG's hilus. The GCL was composed of multiple regular rows of well-organized granular cells with small, rounded granule cell bodies with basophilic cytoplasm, a large vesicular nucleus, and a prominent nucleolus that were arranged in a V-shaped configuration to form the upper (UL) and lower (LL) limbs of the DG. A subgrangular zone (SGZ) was observed beneath the GCL, as well as immature neurons with oval, deeply stained nuclei. Both ML and POL were made up of an eosinophilic neuropil matrix in which glial cells were embedded, as well as normal blood capillaries (Fig. 6A, B).

The GCL of PND-60 rats treated with BPA at a dose of 50 mg/kg/day showed minor disorganization, with the presence of a small number of degraded granular cells. Both ML and POL, on the other hand, exhibited the presence of certain shrunken neurons with pyknotic nuclei and pericellular haloes, as well as flame-like cells with pointed ends. Furthermore, the neuropile developed angiectasis, as well as a reduction in the number of degenerated neuroglial cells (Fig. 6C). The DG of PND-60 animals exposed to a higher dose (125 mg/kg/day) had certain degenerative characteristics compared to those treated with a lower dose, including detectable activation of neuroglial cells, congested blood capillaries, and inflammatory cell infiltration (Fig. 6E).

In the ML and POL of PND 95 rats exposed to BPA at a dose of 50 mg/kg/day, there was an increase in the number of flame-like cells with pointed ends, as well as shrunken neurons with pyknotic nuclei and pericellular haloes. Furthermore, neuroglial cell activity was significantly increased, resulting in the existence of several foci with neurophagia, including the SGZ, which appeared wider and with fewer immature neurons (Fig. 6D). Otherwise, PND 95 rats exposed to higher doses had a marked disarrangement of the GCL, with many apoptotic cells with pyknotic darkly stained nuclei, as well as an apparent increased number of neuroglial cells denoting gliosis and a multiplicity of neurophagia foci localized either in the GCL or in the SGZ. In addition, the neuropil displayed many vacuolations and congested blood vessels. Furthermore, the SGZ appeared wider with a significant reduction in immature neurons compared to rats exposed to a lower dose of BPA (Fig. 6F).

The morphometric analysis revealed a significant (P < 0.05 vs. controls) decrease in the number of pyramidal cells in the PFC in all BPA-exposed groups, with the exception of the PND-60 group treated with BPA at a dose of 50 mg/kg, which showed an insignificant decrease (F_(5,35)_ = 48.547, Fig. 6G). Otherwise, BPA induced a significant decrease in the thickness of GCL of DG (F_(5,35)_ = 59.434, Fig. 6G) and the total number of granular cells in both PFC and DG (F_(5,35)_ = 373.150, 119.115, respectively, Fig. 6H) in all BPA-treated rats. Furthermore, our findings revealed a significantly (P < 0.05 vs. controls) higher count of angiectasis (F_(5,35)_ = 142.336, 178.095, respectively, Fig. 6I), apoptotic cells (F_(5,35)_ = 74.946, 187.956, respectively, Fig. 6J), and relative area of neuroinflammation (F_(5,35)_ = 59.771, 41.087, respectively, Fig. 6K) and neuronal loss (F_(5,35)_ = 78.820, 71.338, respectively, Fig. 6L) in both PFC and DG in BPA-treated rats.

#### Immunohistochemical examination

**Fig. 7:**
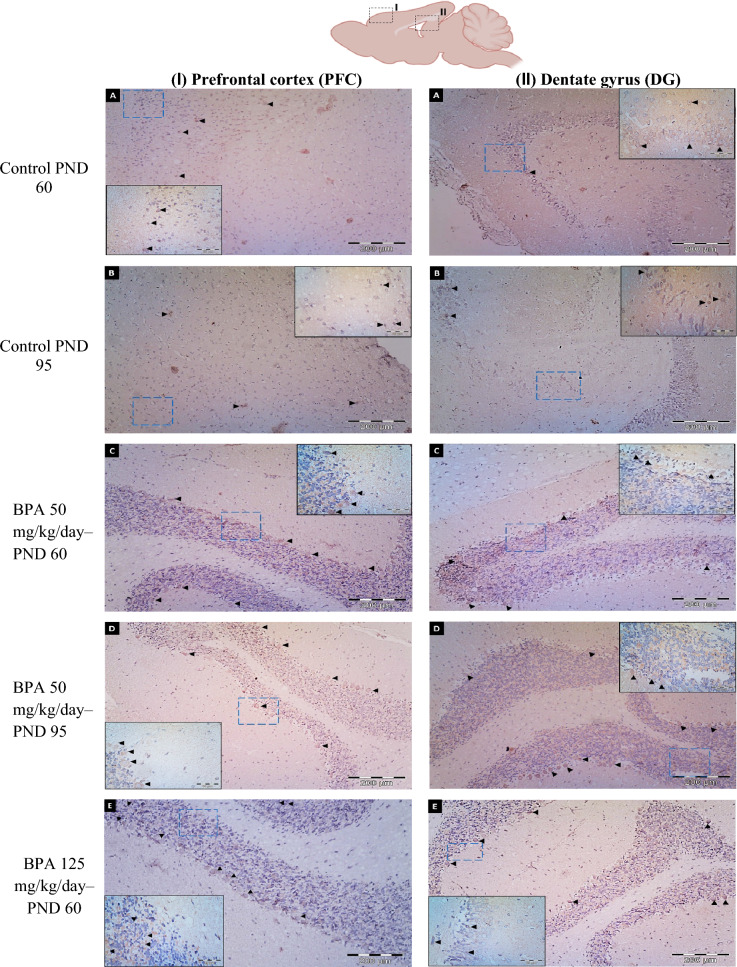

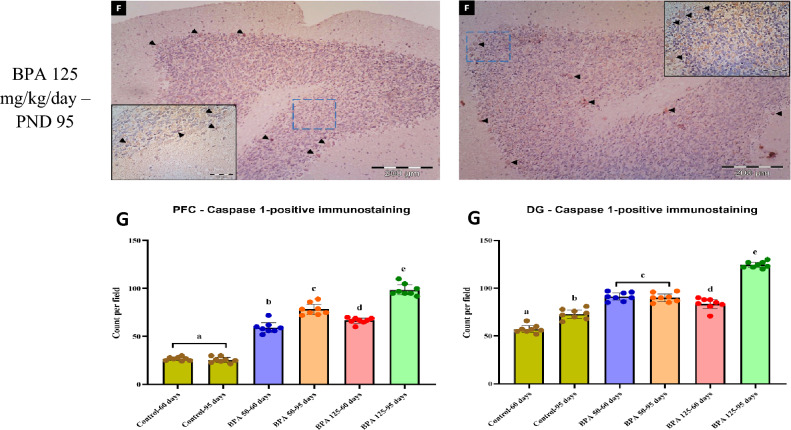
Immunohistochemistry showing the expression of caspase-1 in the PFC and DG of rats in the six experimental groups: **A** Control PND 60, **B** Control PND 95, **C** BPA 50-PND 60, **D** BPA 50-PND 95, **E** BPA 125-PND 60, **F** BPA 125-PND 95. Black arrow heads denote positively brown stained neurons. The blue dashed square indicated the area of magnification.** G** The results are expressed as the mean ± SE (n = 8 fields/group). Groups with different superscript letters means there is a significant change between them at P < 0.05. Statistical significance test for comparison was done by MANOVA, followed by post hoc Tukey’s HSD multiple comparison test

The results of the immunohistochemical examination of caspase-1 expression in the PFC and hippocampus (DG) of different experimental groups are summarized in Fig. 7. In BPA-treated groups, the number of caspase-1 positive immunostainings per field was significantly (P < 0.05) higher in PFC and DG neurons compared to the control groups (F_(5,35)_ = 301.069, 179.537, respectively, Fig. 7G, H).

#### Molecular docking analysis of BPA and target protein structures

**Fig. 8:**
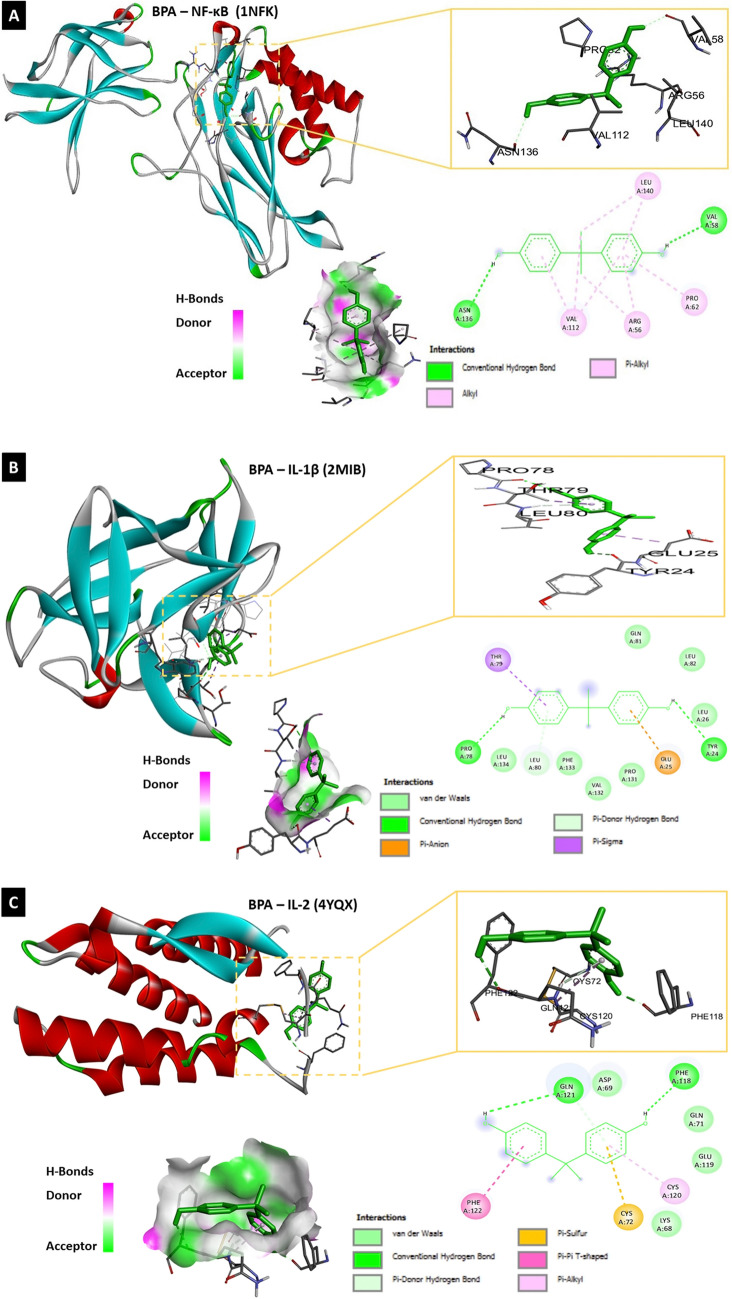

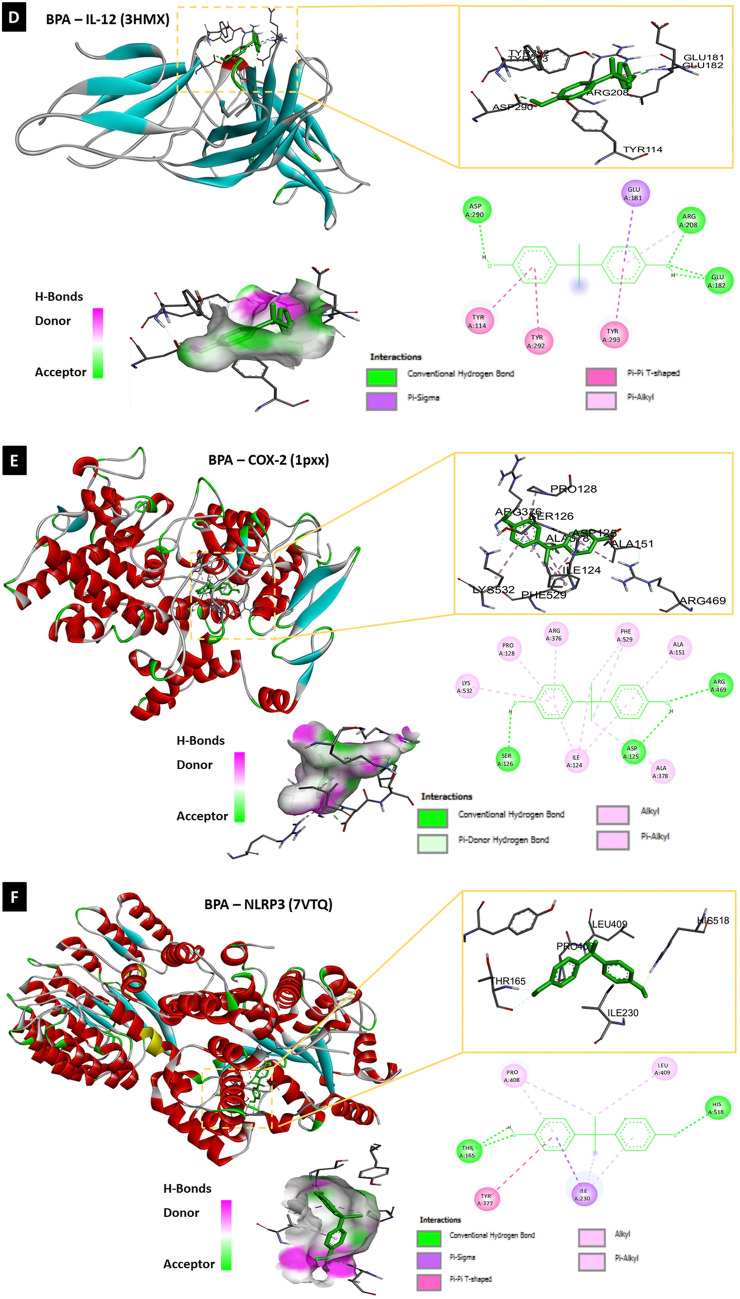

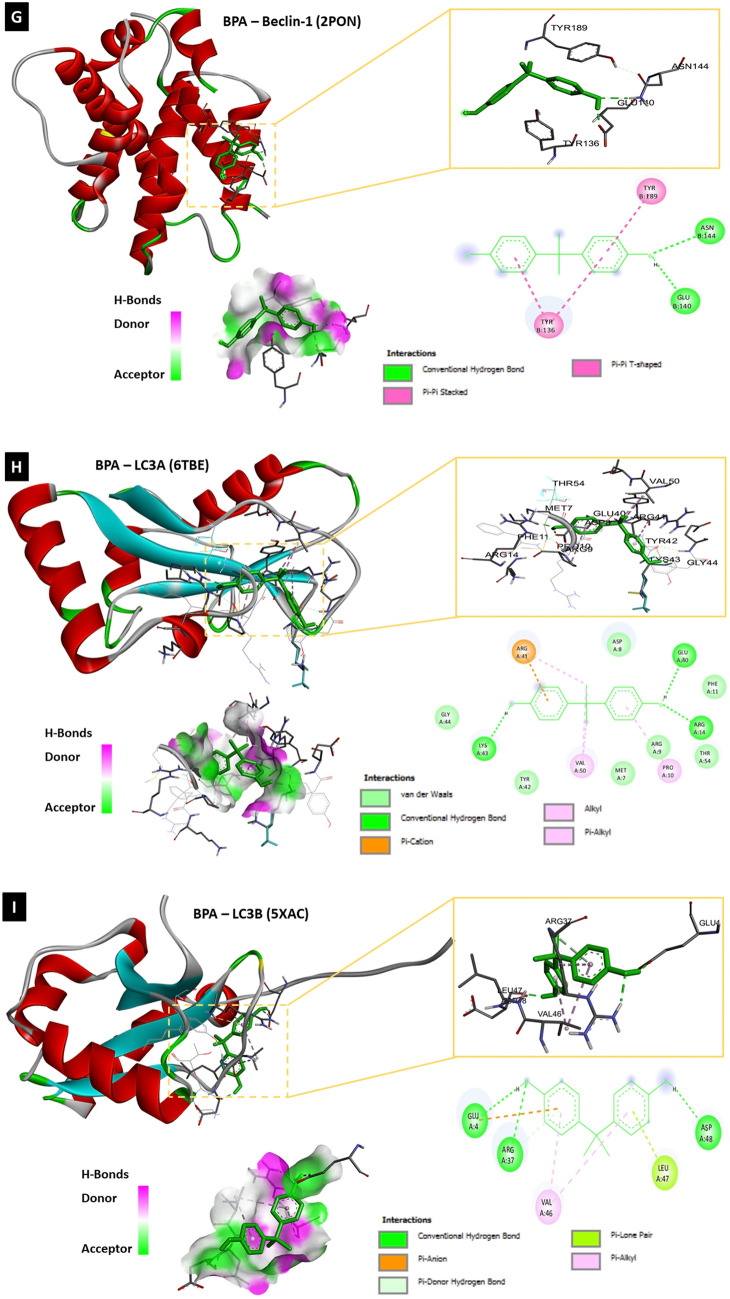

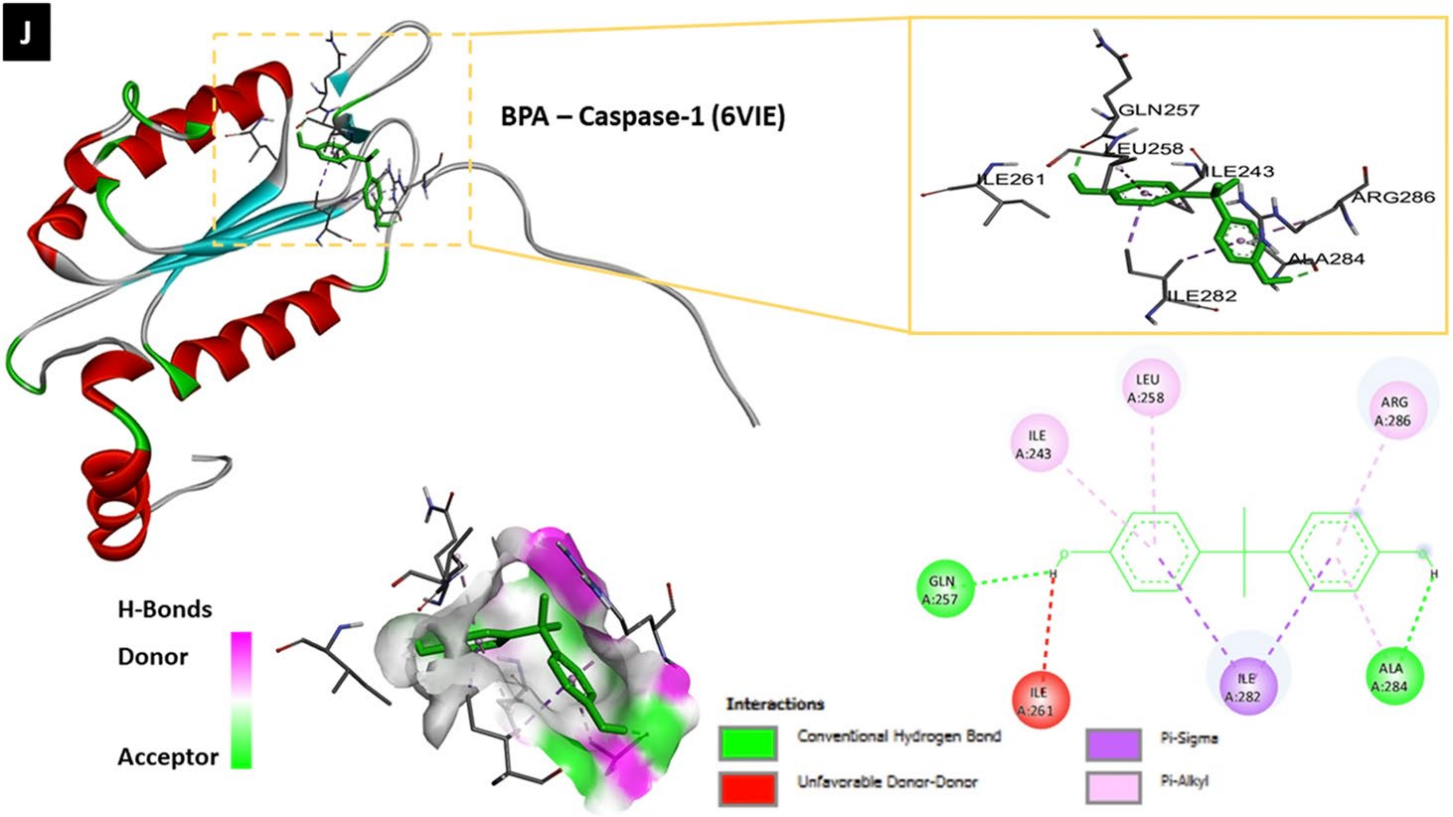
The molecular docking analysis of BPA and neuroinflammatory, autophagic as well as, pyroptotic molecules displaying 2D and 3D binding interactions of BPA [PubChem CID: 6623] against **A** NF-_k_B [PDB ID: 1NFK], **B** IL-1β [PDB ID: 2MIB], **C** IL-2 [PDB ID: 4YQX], **D** IL-12 [PDB ID: 3HMX], **E** COX-2 [PDB ID: 1PXX], **F** NLRP3 [PDB ID: 7vtq], **G** Beclin-1 [PDB ID: 2PON], **H** LC3A [PDB ID: 6TBE], **I** LC3B [PDB ID: 5XAC], **J** Caspase-1 [PDB ID: 6VIE]. The green dotted lines denote hydrogen bonds between ligand and aminoacids, whereas bink/purple dotted lines represent hydrophobic interactions. Electrostatic interactions are shown as orange dotted lines. The red dotted line indicate an unfavorable donor-donor

To determine whether BPA can directly interact with different proteins involved in neuroinflammation, autophagy, and pyroptosis, the binding affinity between BPA and each molecule was assessed using molecular docking analysis with AutoDock 4.2 software and Biovia Discovery Studio 2020. Molecular docking analysis of BPA and the nine target molecules showed that BPA directly interacted with NF-κB, IL-1β, IL-2, IL-12, COX-2, NLRP3, beclin-1, LC3A, LC3B, and caspase-1 (Table 1, Fig. 8A–I).

**Table 1:** Molecular docking analysis of ligand (BPA; PubChem CID: 6623) and target proteins involved in neuroinflammation, autophagy and pyroptosis

Protein ID	Name	RMSD^b^	Binding energy (Kcal/Mol)	Inhibition constant, Ki (nM)	Amino acid involved in interaction
1NFK	NF-κB	40.30	− 6.31	23.53	LEU-140, VAL-58, PRO-62, ARG-56, VAL-112, ASN-136
2MIB	IL-1β	122.01	− 6.66	13.13	TYR-24, GLU-25, LEU-80, PRO-78, THR-70
4YQX	IL-2	138.16	− 5.82	54.60	PHE-118, CYS-120, CYS-72, PHE-122, GLN-121
3HMX	IL-12	170.23	− 7.96	1.46	ARG-208, GLU-182, TYR-292, TYR-293, TYR-114, ASP-290, GLU-181
1PXX	COX-2	67.05	− 7.66	2.43 μM	ALA-151, ARG-469, ASP-125, THR-149, ILE-124, ALA-378, SER-126, LYS-532, PRO-128, ARG-376, PHE-529
7vtq	NLRP3	348.28	− 8.04	9.89	LEU-409, HIS-518, ILE-230, TYR-377, THR-165, PRO-408
2PON	Beclin-1	121.64	− 5.60	78.44	TYR-189, ASN-144, Glu-140, TYR-136
6TBE	LC3A	116.50	− 7.48	3.30	GLU-40, ARG-14, ARG-9, PRO-10, VAL-50, LYS-43, ARG-41
5XAC	LC3B	231.98	− 6.60	14.45	ASP-48, LEU-47, VAL-46, ARG-37, GLU-4
6VIE	Caspase-1	91.9	− 7.11	6.19	ARG-286, ALA-284, ILE-282, ILE-261, GLN-257, ILE-243, LEU-258

#### Behavioral, biochemical, histological, and immunohistochemical correlations

Pearson's linear correlation analysis was utilized to quantitatively corroborate the apparent relationship between the parameters, as shown in Suppl. Table S1. The IL-1β (PFC: r = 0.918, P = 0.010; r = 0.921, P = 0.009; hippocampus: r = 0.868, P = 0.025; r = 0.867, P = 0.025), IL-12 (PFC: r = 0.866, P = 0.026; r = 0.873, P = 0.023; hippocampus: r = 0.884, P = 0.020; r = 0.887, P = 0.019), IL-2 (PFC: r = 0.887, P = 0.018; r = 0.878, P = 0.021; hippocampus: r = 0.877, P = 0.022; r = 0.888, P = 0.018), COX-2 (PFC: r = 0.883, P = 0.020; r = 0.886, P = 0.019; hippocampus: r = 0.899, P = 0.015; r = 0.898, P = 0.015), NLRP3 (PFC: r = 0.863, P = 0.027; r = 0.866, P = 0.026), LC3A (PFC: r = 0.8530, P = 0.0308; r = 0.8597, P = 0.0281), NF-κB (PFC: r = 0.865, P = 0.026; r = 0.868, P = 0.025; hippocampus: r = 0.886, P = 0.019; r = 0.888, P = 0.018), and caspase-1 (PFC: r = 0.880, P = 0.021; r = 0.886, P = 0.019; hippocampus: r = 0.878, P = 0.021; r = 0.880, P = 0.021) were positively correlated with OFT-total and peripheral distance, respectively. Moreover, LC3A was positively correlated with OFT-No. of entry in peripheral (PFC: r = 0.8698, P = 0.0243). While IL-1β, IL-12, COX-2, LC3A, LC3B, NLRP3, NF-κB, and caspase-1 in the PFC were negatively correlated with the center/total time in the OFT (r = − 0.863, P = 0.027; r = − 0.856, P = 0.029; r = − 0.808, P = 0.052; r = − 0.9063, P = 0.0128; r = − 0.986, P = 2.731E-004; r = − 0.808, P = 0.052; r = − 0.889, P = 0.018; r = − 0.840, P = 0.036), as well as the hippocampus IL-12 (r = − 0.837, P = 0.038), and LC3B (r = − 0.928, P = 0.007). Similarly, IL-12 (r = − 0.839, P = 0.037), LC3A (r = − 0.9200, P = 0.0093), LC3B (r = − 0.887, P = 0.019), and caspase-1 (r = − 0.834, P = 0.039) in the PFC were negatively correlated with the OFT entry in the center. Also, beclin-1 in the PFC was negatively correlated with OFT-center distance (r = − 0.935, P = 0.006), as well as positively correlated with OFT entry in the peripheral (r = 0.908, P = 0.012).

The beclin-1 (r = 0.852, P = 0.031) and LC3B (r = 0.905, P = 0.013) in the PFC were positively correlated with the duration to reach exit end in the EPM test. The distance in closed arms of the EPM test was positively correlated with LC3B in the hippocampus (r = 0.839, P = 0.037).

Furthermore, the IL-1β (PFC: r = − 0.825, P = 0.043), IL-12 (hippocampus: r = − 0.824, P = 0.044), IL-2 (PFC: r = − 0.852, P = 0.031; hippocampus: -0.839, 0.037), COX-2 (PFC: r = − 0.859, P = 0.028), LC3B (hippocampus: r = − 0.848, P = 0.033), NLRP3 (PFC: r = − 0.881, P = 0.021; hippocampus: r = − 0.909, P = 0.012), NF-κB (PFC: r = − 0.855, P = 0.030; hippocampus: r = − 0.849, P = 0.033), and caspase-1 (PFC: r = − 0.807, P = 0.052) were negatively correlated with YMT-duration in the home arm, but IL-12 (r = 0.828, P = 0.042) in the PFC, as well as IL-12 (r = 0.835, P = 0.038) and caspase-1 (r = 0.845, P = 0.034) in the hippocampus, were positively correlated with YMT-total arm entries. Similarly, COX-2 and caspase-1 in the hippocampus were positively correlated with the YMT-AAR (r = 0.820, P = 0.045; r = 0.914, P = 0.011, respectively), but beclin-1 (r = − 0.899, P = 0.015) and LC3A (r = − 0.8407, P = 0.0361) in the PFC was negatively correlated with the YMT-Total duration, as well as NLRP3 (r = − 0.813, P = 0.049) and caspase-1 (r = − 0.822, P = 0.045) in the PFC were negatively correlated with the YMT-duration in the novel arm.

The behavioral variables of the OFT, EPM, and Y-maze tests were highly correlated with the morphometric results of histopathology in the PFC and DG. The relative area of neuronal loss in the PFC was correlated with OFT-center distance (r = − 0.820, P = 0.046), OFT-entry in the center (r = − 0.912, P = 0.011), OFT-entry in peripheral (r = 0.839, P = 0.037), EPM-duration to reach exit end (r = 0.929, P = 0.007), and YMT-total duration (r = − 0.868, P = 0.025). However, the relative area of neuronal loss in the DG was positively correlated with OFT-total distance (r = 0.963, P = 0.002), OFT-peripheral distance (r = 0.963, P = 0.002), and YMT-AAR (P = 0.880, r = 0.021). Angiectasis was correlated with the OFT-total distance (PFC: r = 0.875, P = 0.022; DG: r = 0.894, P = 0.016), OFT-peripheral distance (PFC: r = 0.878, P = 0.021; DG: r = 0.900, P = 0.015), OFT-center/total time (PFC: r = − 0.841, P = 0.036; DG: r = − 0.883, P = 0.020), YMT-duration in the home (PFC: r = − 0.856, P = 0.030), OFT-entry in peripheral (DG: r = 0.848, P = 0.033), YMT-total duration (DG: r = − 0.843, P = 0.035).

The relative area of inflammatory cell infiltration was correlated with the OFT-total distance (PFC: r = 0.941, P = 0.005; DG: r = 0.900, P = 0.014), OFT-peripheral distance (PFC: r = 0.944, P = 0.005; DG: r = 0.906, P = 0.013), OFT-center/total time (PFC: r = − 0.829, P = 0.041), OFT-entry in the center (DG: r = − 0.872, P = 0.023), OFT-entry in peripheral (DG: r = 0.819, P = 0.046), EPM-duration to reach exit end (DG: r = 0.829, P = 0.042), and YMT-total duration (DG: r = − 0.850, P = 0.032).

Granular cell counts were correlated with OFT-total distance (PFC: r = − 0.820, P = 0.046; DG: r = − 0.805, P = 0.053), OFT-peripheral distance (PFC: r = 0.828, P = 0.042; DG: r = − 0.809, P = 0.051), OFT-center distance (PFC: r = 0.823, P = 0.044), OFT-entry in the center (PFC: r = 0.948, P = 0.004), OFT-entry in peripheral (DG: r = − 0.816, P = 0.048), OFT-center/total time (PFC: r = 0.812, P = 0.050), EPM-duration to reach exit end (PFC: r = − 0.875, P = 0.023), and YMT-duration in the novel (PFC: r = 0.885, P = 0.019), YMT-home arm visits (DG: r = 0.916, P = 0.010), YMT-total arm entries (DG: r = − 0.975, P = 0.001), and YMT-total duration (DG: r = 0.904, P = 0.013).

Pyramidal cell counts in the PFC were correlated with OFT-entry in the center (r = 0.892, P = 0.017), EPM-duration to reach exit end (r = -0.820, P = 0.046), EPM-distance in open arms (r = 0.816, P = 0.048), YMT-duration in the novel arm (r = 0.897, P = 0.015).

Apoptotic cell counts were correlated with OFT-total distance (PFC: r = 0.815, P = 0.048; DG: r = 0.845, P = 0.034), OFT-peripheral distance (PFC: r = 0.824, P = 0.044; DG: r = 0.853, P = 0.031), OFT-center distance (PFC: r = − 0.868, P = 0.025; DG: r = − 0.832, P = 0.040), OFT-entry in the center (PFC: r = -0.948, P = 0.004; DG: r = -0.936, P = 0.006), OFT-entry in peripheral (PFC: r = 0.848, P = 0.033), EPM-duration to reach exit end (PFC: r = 0.889, P = 0.018; DG: r = 0.821, P = 0.045), YMT-duration in the novel arm (PFC: r = − 0.844, P = 0.035; DG: r = − 0.869, P = 0.025), and YMT-total duration (PFC: r = − 0.870, P = 0.024; DG: r = − 0.823, P = 0.044).

The thickness of granular cell in the DG was correlated with OFT-center distance (r = 0.924, P = 0.008), OFT-entry in the center (r = 0.965, P = 0.002), OFT-center/total time (r = 0.839, P = 0.037), EPM-duration to reach exit end (r = − 0.924, P = 0.008), EPM-distance in open arms (r = 0.855, P = 0.030), YMT-duration in the novel arm (r = 0.867, P = 0.025). The data that support the findings of this study are available upon reasonable request from the corresponding author.

#### Multivariate analysis

A multivariate study of inflammatory markers (IL-1β, IL-12, IL-2, COX-2, and NF-κB) in the PFC produced the centroid PCO ordination graph shown in Fig. 4K. The PCO axis 1 explained 91.5% of the overall variation in data, separating the controls 60 and 95, as well as BPA 50–60, and BPA 50 and 125 at PND 95 on the positive side of the axis and BPA 125–60 on the negative. By separating all BPA dosages and ages (on the right side of the axis) from controls (on the left side of the axis), the PCO axis 2 explained 7.1% of total data variation. Postnatal rats from the control and BPA 50–60 groups had greater levels of IL-2, IL-12, and NF-κB; rats exposed to BPA 50 and 125 at PND 95 had higher correlations with COX-2,and rats exposed to BPA 125 at PND 60 had higher levels of IL-1β.

A multivariate study of inflammatory markers in the hippocampus produced the centroid PCO ordination graph shown in Fig. 4L. The PCO axis 1 explained 87.3% of the overall variation in data, with BPA 50 and BPA 125 at PND 60 on the positive side and controls 60 and 95, BPA 50 and 125 at PND 95 on the negative side. By differentiating all BPA doses and ages (on the right side of the axis) from the controls (on the left side of the axis), PCO axis 2 explained 12.5% of the total data variation. Postnatal rats exposed to BPA 50 and 125 at PND 95 had greater IL-1β levels; rats exposed to BPA 50–60 had higher IL-2, IL-12, and NF-κB levels, and rats exposed to BPA 125–60 had higher COX-2 levels.

As a result of using multivariate analysis of autophagy markers (NLRP3, beclin-1, LC3A, and LC3B) in the PFC, the centroid PCO ordination graph is presented in Fig. 5G. The PCO axis 1 explained 86.5% of the total variation in data, separating control 60 and BPA 125 at PND 95 on the positive side, and control 95, BPA 50 at PND 60 and PND 95, and BPA 125 at PND 60 on the negative side. BPA 50–95, BPA 125–95, and BPA 125–60 (on the right side of the axis) were distinguished from controls 60 and 95, and BPA 50–60 (on the left side of the axis) by the PCO axis 2 (12.6%). Postnatal rats from the control 60 had greater LC3B levels; rats exposed to BPA 50 and 125 at PND 95 had better correlation with LC3A levels; rats from the control 95 and exposed to 50 and 125 doses of BPA at PND 60 had a better correlation with beclin-1 levels, and rats exposed to BPA 50 and 125 at PND 95 had better correlation with NLRP3 levels.

The centroid PCO ordination graph is shown in Fig. 5H as a result of a multivariate analysis of autophagy markers in the hippocampus. The PCO axis 1 explained 77.6% of the overall variation in data, separating control 95, BPA 50 at PND 60 and 95, and BPA 125 at PND 60 and 95 on the positive side, and control 60 on the negative side. The PCO axis 2 explained 13.4% of the total data variation, separating the control 60, as well as BPA 125–60, BPA 50–95, and BPA 125–95 (on the right side of the axis) from control 95 and BPA 50–60 (on the left side of the axis). Postnatal rats from the control 60 had greater NLRP3 levels; rats exposed to BPA 50–60 showed a better connection with beclin-1 levels, rats exposed to BPA 50–95 showed a better correlation with LC3A levels, and rats exposed to BPA 50 and 125 at PND 95 and BPA 125–60 had higher LC3B levels.

## Discussion

BPA exposure during early developmental stages such as embryonic, infant, and childhood stages has been linked to adverse neurodevelopmental outcomes such as anxiety-like behavior, cognitive dysfunction, and memory impairments, which have been well supported by findings from both epidemiological studies and animal behavioral tests [29, 30]. Anxiety disorders are among the most common psychiatric diseases, and because they emerge relatively during early-life, they may be chronic or recurrent throughout life, resulting in a considerable disease burden [31]. In the current study, we revealed a link between postnatal BPA exposure at doses of 50 and 125 mg/kg/day, age, and the manifestation of psychiatric symptoms such as anxiety-like behaviors associated with cognitive and memory deficits in male rats.

Although many behavioral studies conflict, there appears to be a link between perinatal BPA exposure and alterations in stress-related behavioral endpoints. In this work, we looked at male rats' behavioral reactions at two ages: adolescence (PND 34–60) and adulthood (PND 65–95). The two age points chosen for our study represent the pinnacle of adolescence and complete sexual development. We found that anxiety was exhibited differently during distinct periods of late postnatal development. BPA-exposed male adolescent and adult rats in the OFT showed a significant increase in the number of laps but a significant decrease in the distance travelled and the number of entries to the central zone. However, the distance travelled and the number of entries to the peripheral zone were found to be significantly increased. These behavioral changes corroborate previous findings by Zhou et al. [32] and Sasaki et al. [33], who demonstrated that BPA exposure during early developmental stages caused long-term anxiety-related behaviors, which may be associated with the neonatal activity of sex hormones, which are essential for dopaminergic pathways, which are essential for controlling locomotor activities. The literature on BPA’s effects on the brain and behavior remains incongruent. BPA's modest binding affinity for estrogen receptors ERα and ERβ, as well as its agonist and antagonist properties, could explain this [34]. Rather, certain effects may be mediated by non-classical interactions with the estrogen receptor. Alternatively, one could explain the results to a non-monotonic property of BPA, such that low-dose effects develop, fade at mid-range, and possibly reappear at higher doses. The increase in distance travelled in the central zone is attributed to an anxiolytic-like condition; nevertheless, distance walking in the middle zone has also been used to assess exploratory/locomotor activity. Thus, it appears that high concentrations of prenatal BPA exposure were sufficient to induce an anxiolytic character in juveniles [34]. Furthermore, the significantly lower number of entries into the periphery for BPA 125–95 compared to other BPA-treated groups could be attributed to anxiety-like behavior on classic behavioral tests gradually reducing in rats as they progress from adolescence to adulthood. As a result, adolescent rodents may provide useful information on the mechanisms by which vulnerability to anxiety disorders develops from adolescence to adulthood in humans [23, 35]. This was consistent with our findings in Pearson's correlation, where the relative area of neuronal loss in the PFC was correlated with OFT-center distance, OFT entry in the center, and OFT entry in the peripheral; however, the relative area of neuronal loss and inflammatory cell infiltration, angiectasis, apoptotic cell counts, and thickness of granular cell was positively correlated with OFT-total distance, OFT-peripheral distance, OFT-entry in the center, OFT-center/total time, and OFT-entry in peripheral. Furthermore, it could be triggered by neuroinflammation and autophagy produced by the NF-κB/NLRP3/Caspase-1 signaling pathway. NF-κB, IL-1β, IL-12, IL-2, COX-2, NLRP3, and caspase-1 were all found to be positively correlated with OFT-total and peripheral distance. In the PFC, NF-κB, IL-1β, IL-12, COX-2, NLRP3, LC3A, LC3B, and caspase-1 were negatively correlated with the center/total time in the OFT. Similarly, IL-12, LC3A, LC3B, and caspase-1 in the PFC were found to be negatively correlated to OFT entry in the center. Furthermore, beclin-1 and LC3A in the PFC were found to be negatively correlated with OFT-center distance and positively correlated with OFT-entry in the peripheral.

Anxiety is typically assessed on the EPM as a decreased tendency for exploration of the open arms and/or increased visits to the closed arms [36]. When the EPM was used to investigate whether ageing affects anxiety-like behaviors in mice, the results were mixed, with numerous studies reporting inconsistent findings. For example, a study of a large number of C57BL/6J mice up to 12 months old found that while the distance travelled in the open arms decreases with age, time spent and percentage entries in the open arm significantly increase, implying a decrease in anxiety-like behavior from young to middle age, with the same conclusions found in rats [37]. In this study, BPA-exposed postnatal male rats had increased distance in the closed arms and decreased distance in the open arms, which is reflected in an overall decreased activity in the EPM, including total distance and time spent in the open arm/total time spent for both arms, particularly in the PND 95 rats, combined with an increased duration needed to reach the exit end. These findings are consistent with those of Wang et al. [30], who demonstrated that BPA exposure resulted in the development of anxiogenic depressive-like behaviors in which BPA caused pyramidal neuron dysfunction and microglia activation in the PFC [30]. The incremental changes in rats' performance on the EPM correspond to changes in adrenal hormone production at different ages. For example, the HPA axis develops from hyperresponsiveness in prepubertal animals to decreased responsiveness in adulthood, as well as prolonged corticosterone production in response to stress when compared to adults [38]. A rising number of studies have found that stress has a more pronounced and long-lasting effect on adolescent rodents than on adults [39, 40]. Furthermore, human adolescence is associated with increased baseline and stress-induced HPA axis activity [41]. Moreover, a small decrease in the distance travelled to closed arms of BPA 50–60 because anxiety was exhibited differently during distinct periods of late postnatal development. The adolescent stage in rats begins about PND 28, correlating with growing circulating gonadal hormone levels. Rats are normally considered sexually mature when they reach PND 60. However, male testes continue to develop into young adulthood, and testosterone levels peak at about PND 70 before decreasing to adult levels. As a result, adolescence is believed to be a time when the growing stress axis is more susceptible to perturbations, and stress exposure at this stage of life may have long-term consequences for human mental health [35]. These findings are consistent with the conclusions reached by Albani et al. [42], who found that late preweaning rats have distinct sensitivities to environmental cues than adults while also displaying less fear of open areas, even within their home cages. Overall, the data support the hypothesis that juvenile rats’ EPM behavior is the result of overlapping developmental trajectories for numerous brain systems involved in sensorimotor skill, anxiety, and risk evaluation. This was consistent with our findings in Pearson’s correlation, where the relative area of neuronal loss and inflammatory cell infiltration, angiectasis, apoptotic cell counts, and thickness of granular cells in the DG was positively correlated with EPM-distance in open arms and duration to reach the exit end. Moreover, it could be caused by the beclin-1 and LC3B as they were positively correlated with the duration to reach the exit end and the distance in closed arms of the EPM test.

The classic spatial memory Y-maze model, which is dependent on the hippocampus to explore spatial surroundings, was used to investigate the effect of BPA exposure on rat cognitive behaviors. Our results from the Y-maze test showed that BPA-treated rats spent less time exploring the novel arm compared to both the home and familiar arms. These findings substantially confirm the data that BPA exposure throughout childhood disrupts hippocampus-dependent spatial memory consolidation [43]. Previous studies have found that an imbalance of dopamine and GABA neurotransmitters can all contribute to cognitive impairment. As a result, BPA affects cognitive impairment via altering NMDA receptor activity. Taken together, the behavioral findings imply that the juvenile-age phase between adolescence and adulthood is vulnerable to BPA toxicity and that exposure to BPA during this phase might lead to spatial memory problems later in life, as we found in our earlier research [44]. This was consistent with our findings in Pearson's correlation, where angiectasis, the relative area of neuronal loss and inflammatory cell infiltration, angiectasis, apoptotic cell counts, and thickness of granular cell was correlated with YMT- duration in the novel, duration in home, total duration, home arm visits, and total arm entries. Furthermore, it could be triggered by neuroinflammation and autophagy produced by the NF-κB/NLRP3/Caspase-1 signaling pathway. NF-κB, IL-1β, IL-12, IL-2, COX-2, LC3B, NLRP3, and caspase-1 were negatively correlated with YMT-duration in the home arm, but IL-12 in the PFC, as well as IL-12 and caspase-1 in the hippocampus, were positively correlated with YMT-total arm entries. Similarly, the COX-2 and caspase-1 in the hippocampus were positively correlated with the YMT-AAR, but the beclin-1 in the PFC was negatively correlated with the YMT-Total duration, as well as NLRP3 and caspase-1 in the PFC, were negatively correlated with the YMT-duration in the novel arm.

**Fig. 9:**
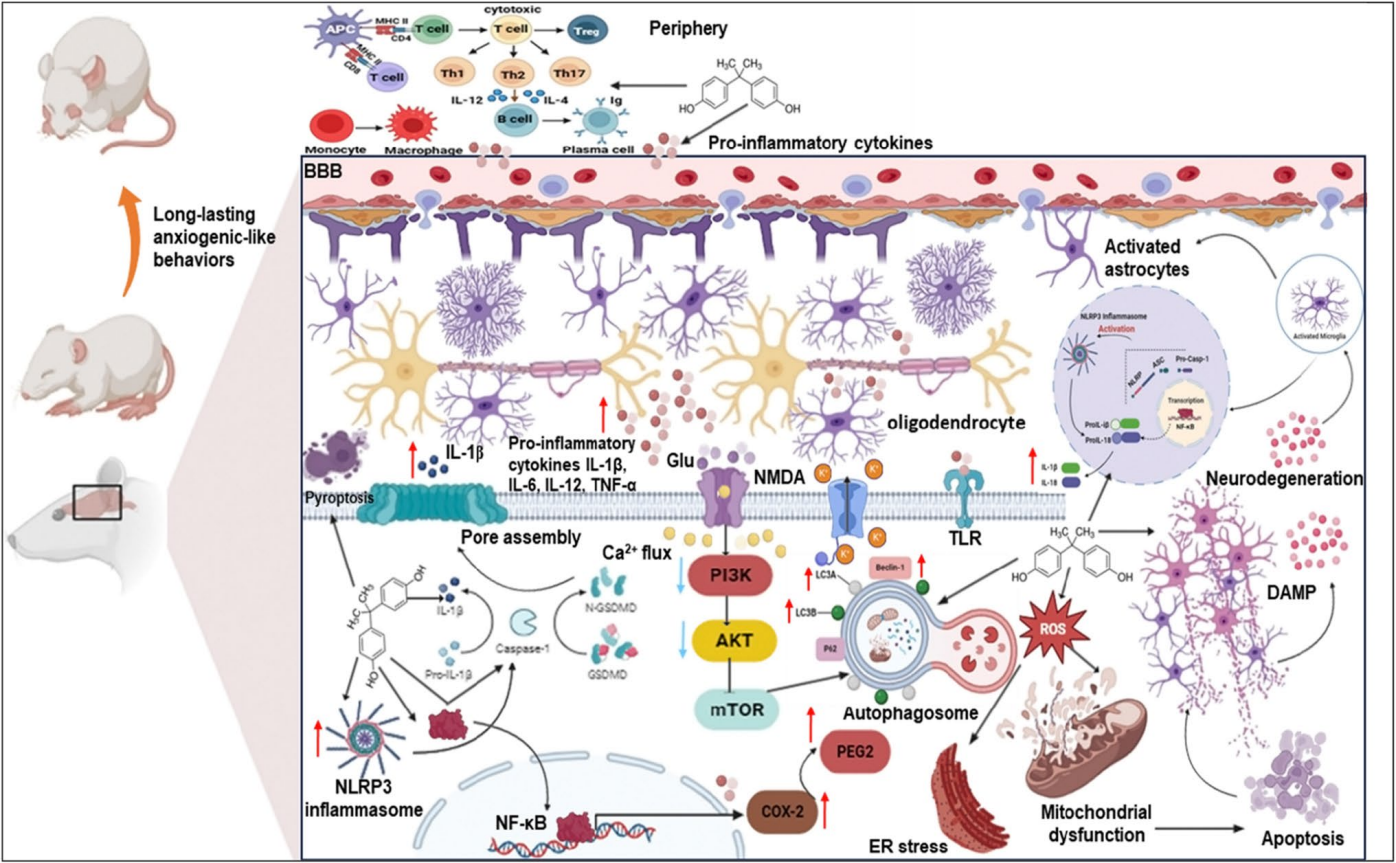
Schematic illustration showing the influence of BPA on the cross-talk between neuroinflammation and autophagy mediated initiation of pyroptosis and progression of anxiety-related behaviors in postnatal male rats. BPA exposure resulted in a remarkable elevation in the level of proinflammatory cytokines particularly IL-2 and IL-12 that are significant for differentiation and function of T-cells. The Canonical NLRP3 inflammasome activation requires two steps: the first is priming step and the second is activation step. In the priming step, toll-like receptors (TLR) stimulation promotes the transcription and expression of NLRP3 and pro-IL-1β through NF-κB. Subsequently, various DAMPs and PAMPs induce the activation step by initiating numerous cellular and molecular cascades, including K^+^ efflux, Ca^2+^ influx, mitochondrial dysfunction, reactive oxygen species (ROS) release, and lysosomal disruption. The NLRP3-dependent self-cleavage and activation of pro-caspase-1 self-cleavage and activation leads to the maturation of the pro-inflammatory cytokines IL-1β and IL-18. Additionally, gasdermin D (GSDMD) is cleaved by activated caspase-1, releasing its *N*-terminal domain, which then integrates into the cell membrane to create pores. These pores allow the release of cellular contents, including IL-1β and IL-18, and trigger pyroptosis. BPA exposure caused a significant elevation in the expression level of NF-κB, IL-1β, NLRP3 and caspase-1. The release of DAMPs and PAMPs is a combined by an increase in extracellular glutamate levels, which further leads to excitotoxic neuron damage through NMDA receptors. Furthermore, glutamate-mediated Ca^2+^ influx occurs through NMDA receptors. mTOR is a downstream target of PI3K and AKT pathway which could be downregulated by apoptotic signals, therefore, induction of autophagy by inhibition of the mTOR may result in dysregulated autophagic machinery led to neuronal degeneration. Damaged and dying neurons secrete DAMPs besides prevalence of cytokines all together lead to activation of microglia via binding to cell surface receptors (e.g. TLR), resulting in activation of the NF-κB pathway. This activation induces a signaling cascade, causing enhancement in the expression of cytokines such as pro-IL-1β and pro-IL-18 as well as NLRP3. Autophagy begins with the formation of double-membrane vesicles known as autophagosomes. Autophagosomes combine with lysosomes to generate autophagolysosomes, which degrade their contents into basic molecules that can be recycled for reuse. The elevated beclin-1, LC3A, and LC3B levels by inhibiting the PI3K/Akt/mTOR signaling pathway, triggering autophagy in brain tissue. Once microglia get activated, they become polarized to an M1, pro-inflammatory phenotype and secrete pro-inflammatory cytokines and factors as well as, activating astrocytes. Activation and dysfunction of astrocytes contributes to BBB, allowing infiltration of different immunological cells. *Note* At the end of each line, the arrowhead indicates activating the neural projection, while short line for inhibition

BPA causes neurotoxicity through a variety of pathways, including neuroinflammation, mitochondrial dysfunction, the formation of reactive oxygen species (ROS), and protein degradation, which ultimately leads to apoptosis [45]. NF-κB is a transcription factor family member that is thought to be a playmaker in suppressing inflammatory reactions and a crucial factor in linking cytoplasmic and nuclear signal transduction. NF-κB is maintained in the cytoplasm of unstimulated cells via conjugation to IkB-α. Once activated, NF-κB promotes nuclear translocation by phosphorylation of its endogenous inhibitor IkB-α, resulting in the regulation of gene expression of specific cytokines such as COX-2, iNOS, and TNF-α [46; Fig. 9]. The present study found that postnatal BPA intoxication significantly increased the level of the neuroinflammatory mediator; NF-κB. Our findings are consistent with those of Zhu et al. [47] and Elbakry et al. [48], who found that BPA dramatically increased the expression level of phosphorylated NF-κB p65, supporting the role of NF-κB in the inflammatory response.

COX-2 is located in excitatory terminals and postsynaptic dendrites of neurons in the neocortex, amygdala, hippocampus, and dorsal horn of the spinal cord [49]. COX-2 level is significantly increased in astrocytes and microglia during a neuroinflammatory response [50]. In the current study, we found that postnatal BPA exposure increases COX-2 levels in both the PFC and the DG and exacerbates neuroinflammation. Song et al. [51] demonstrated that BPA caused an increase in the phosphorylation rate of the extracellular signal-regulated kinase (ERK) 1/2, which resulted in the induction of COX-2 production, implying the participation of both the NF-κB and mitogen-activated protein kinase (MAPK) signaling pathways. Furthermore, Abdou et al. [52] demonstrated that increased generation of ROS/RNS as well as pro-inflammatory cytokines increased COX-2 mRNA expression in the brains of BPA-treated rats.

IL-2 is a pleiotropic cytokine produced mostly by T-cells that play a key role in the initiation and control of the cellular immune response in the periphery. It is also found in the CNS, where it works as a neuromodulator in certain brain areas such as the PFC, hippocampus, cerebellum, hypothalamus, locus coerulus, septum, and striatum of humans and other mammals [53]. In our study, IL-2 was noticed to be increased in the PFC and hippocampus of BPA-exposed rats, these findings are in concord with those of Gilio et al. [54] who demonstrated that elevated levels of pro-inflammatory cytokine IL-2-induced anxiogenic depressive-like behaviors which could be referred to capability of IL-2 to interfere with the NMDA receptor functions and to cause inhibition in the long-term potentiation induction and maintenance [55]. Furthermore, increased levels of IL-2 have been linked to the degradation of the tryptophan enzyme, a precursor to serotonin synthesis, via the indoleamine 2,3-dioxygenase immune pathway, producing neurodegeneration and initiating anxiogenic and depressive disorders [56].

IL-12 is a key inflammatory mediator produced by innate cells such as dendritic cells and macrophages that promotes the development of naive T cells into T helper type 1 (Th1) cells producing IFN-γ [57]. BPA treatment increased IL-12 production in both juvenile and adult-age stage rats exhibiting anxiety-like behaviors. These findings are consistent with those of Tong et al. [58], who showed that a cluster of pro-inflammatory cytokines, including IL-12, has a role in the severity of anxiety and depression. The effect of BPA exposure on IL-12 productivity in the CNS has not previously been studied, but Alizadeh et al. [59] proposed that the mild estrogenic action of BPA could trigger a shift in immune response towards Th1 dominance via increased IL-12 synthesis in BPA-treated rats.

Pyroptosis is a highly inflammatory form of programmed cell death that requires the NLRP3 inflammasome to be activated [60]. NLRP3 inflammasomes are crucial components of immunological signaling processes that regulate the maturation of pro-caspase 1 and the production of pro-inflammatory cytokines IL-18 and IL-1β in response to PAMPs and DAMPs [61; Fig. 9]. Interestingly, activation of the NLRP3 inflammasome has been linked to the beginning and progression of CNS disorders such as anxiety and major depression [62]. In our results, we observed that BPA significantly upregulated the level of IL-1β and the expression of caspase-1 immunoreactive cells in both the PFC and the hippocampus, as well as the activation of the NLRP3 inflammasome. There was currently minimal evidence indicating that the NLRP3 inflammasome played a critical role in the toxic effects of BPA on the neurological system. Because NLRP3 levels increase in both age groups, our findings point to an age-independent influence on BPA exposure. Pirozzi et al. [63], found that daily BPA exposure dramatically accelerated the progression of liver fibrosis mediated by the NLRP3 inflammasome in adult obese mice. Furthermore, Zhang et al. [64] demonstrated that BPA triggered the pyroptotic death of osteocytes via the ROS/NLRP3/Caspase-1 signaling pathway.

Although autophagy can induce cellular malfunction and cell death, it also plays an important role in cleaning and recycling components of aged or damaged cells [65]. Autophagy begins with the formation of double-membrane vesicles known as autophagosomes. Autophagosomes combine with lysosomes to generate autophagolysosomes, which degrade their contents into basic molecules that can be recycled for reuse [66]. There is disagreement on whether autophagy serves as a pro-survival response in neurodegeneration [67]. Thus, whether autophagy is useful or destructive appears to be determined by many types of external stimuli or the degree of autophagy, which may serve as a master switch between cell death and survival. It has been demonstrated that numerous critical proteins, including Beclin-1 and LC3, regulate the autophagy pathway [68]. Beclin-1 is a key player in the localization of autophagic proteins to a pre-autophagosomal structure, as well as in the regulation of autophagy in many physiological and pathological processes [69]. Our study showed that postnatal BPA exposure resulted in a considerable increase in beclin-1 levels in the PFC and hippocampus. These results were supported by a study by Park et al. [66] in which BPA triggered autophagy, which altered the cellular survival of B cells by increasing the nuclear translocation of Nrf2 via ROS generation, leading to its binding to the beclin-1.

LC3 is a key precursor for monitoring autophagosome development. During autophagy, cytoplasmic LC3A transforms into autophagosomal membrane-bound LC3B, resulting in a punctate pattern of LC3 expression [65]. The production of autophagosomes is mediated by changes in autophagy-related genes and microtubule-associated protein 1A/1B-light chain 3 (LC3) [66; Fig. 9]. Previous studies have shown that toxic chemicals increase Beclin-1 and LC3 expressions, leading to neurotoxicity through autophagy [70–73]. In this study, BPA exposure increased the level of LC3A and LC3B in both brain regions in a way similar to that of BPA-exposed mouse neuroblastoma N2a cells [74]. This clearly shows that BPA inhibits autophagic flow. A study by Gur et al. [65] illustrated that acrylamide elevated beclin-1, LC3A, and LC3B levels by inhibiting the PI3K/Akt/mTOR signaling pathway, triggering autophagy in brain tissue. Thus, an increase in the amount of the lower molecular-weight LC3B protein can be recognized, and an increase in the LC3B/A ratio can be utilized to identify autophagy that is upregulated [75]. As such, we used Beclin-1 and LC3 as autophagy indicators. Moreover, the LC3B/LC3A ratio was significantly decreased in the PFC of all groups, whereas no significant changes showed in BPA 50–60. Otherwise, the LC3B/LC3A ratio was significantly increased in the hippocampus of BPA 125 at PND 60 and PND 95, while BPA 50 showed no significant changes at both ages. These findings may also help to clarify one of the likely mechanisms underlying age and dose differences in BPA exposure. It is nevertheless important to remember, however, that if stress conditions in a tissue become sufficiently severe, autophagy-related cell death can become pathologically excessive [76]. During the maturation process, autophagy-related gene (Atg) 4 cleaves LC3, resulting in cytosolic LC3A, which is converted to LC3B after lipidation and binds to the autophagosome membrane, which has a high affinity for autophagic vesicles, allowing them to engulf debris and fuse with lysosomes [76]. The half-life of LCB3B is extremely brief, and Atg4 may cleave LC3B at the post-ischemic events, releasing the LC3A form [77, 78]. Thus, the increased LC3B-II/LC3B-I ratio indicated more intense autophagy production in the hippocampus of the BPA 125 group. Significantly, a region-related decline of function in the PFC of BPA-treated groups could be related to progressively increased levels of LC3A (inactive form), a deficit that promotes inflammasome and NF-κB activation, exacerbating tissue destruction and region-related physical deterioration [79]. This is concomitant with the study of Gao et al. [80], who found that BPS inhibited autophagy by increasing p-mTOR/mTOR, subsequently down-regulating autophagy flux-related biomarkers (LC3B/LC3A and Beclin-1) and impeding the degradation of autophagy substrate p62.

The neuronal cell injury caused by BPA was evident in this work by the remarkable microscopic changes in the architecture of the PFC and the DG of the hippocampus upon H&E examination, indicating that BPA may have cytotoxic action. Our findings revealed that BPA exposure reduced the number of neurons while increasing the number of glial cells in the PFC and DG. These findings are consistent with the findings of Habibi et al. [81], who revealed that BPA injection increased the number of glial cells, implying that this is due to rapid hypertrophy or proliferation of these cells. Previous studies have demonstrated that BPA promotes mitochondrial damage, resulting in less energy and increased ROS. Furthermore, BPA was found to trigger apoptosis in several types of cells by upregulating Bax expression and downregulating Bcl-2 expression level, which is then transported to the mitochondria and increases the activity of caspases-3, -8, and -9. When the expression rate and activity of caspases change, the machinery of apoptosis is activated [82]. This could account for the appearance of black neurons with markedly condensed cytoplasm and nucleoplasm.

Our light microscopic results further demonstrated that the degenerative changes in DG neurons caused by BPA were largely dependent on the age of animals or dose variation. The degenerative changes were observed as shrinkage and the formation of a large vacuolated pericellular space (halo). The existence of vacuolated pericellular spaces was caused mostly by neuron shrinkage and withdrawal of their cytoplasmic processes as a result of the disintegration of the cytoskeletal elements that support these cells [19, 83]. Estrogen has been shown to influence both cellular proliferation and survival in adult animals by neural stem cells situated in the subgranular zone (SGZ) of DG [84]. Interestingly, during the early postnatal period, BPA was observed to lower the expression level of ER-α in the developing hippocampus of male rats. This could explain the significant decrease in immature neurons in the SGZ, implying that BPA could alter ER-α production and phosphorylation while interrupting nuclear translocation during this developmental stage [19, 69].

## Conclusion

Taken together, our findings exhibit that early-life exposure to BPA causes anxiety-like behaviors, and cognitive and memory deficits in male rats during a critical time of neural development. These behavior alterations are linked to the interaction of neuroinflammation and pyroptosis. The results in this study show that BPA promotes neuroinflammation by increasing pro-inflammatory cytokines such as NF-κB, IL-1β, IL-2, and IL-12, in addition to enhanced COX-2 level and NLRP3 activation. Furthermore, BPA enhanced autophagic flux by increasing the level of LC3A, LC3B and beclin-1 in the PFC and hippocampus of PND 60 and PND 95 male rats, resulting in the initiation of pyroptotic cell death via the NF-κB/IL-1β/NLRP3/Caspase-1 pathway. These findings could help us understand the process of anxiety development throughout the early stages of life induced by BPA exposure.

## Supplementary Information

Below is the link to the electronic supplementary material.

Supplementary file1 (XLSX 36 KB)

## Data Availability

The data The datasets generated during and analyzed during the current study are available from the corresponding author on reasonable request.
